# A robust Fe-based heterogeneous photocatalyst for the visible-light-mediated selective reduction of an impure CO_2_ stream†

**DOI:** 10.1039/d4sc02773f

**Published:** 2024-06-19

**Authors:** Topi Ghosh, Peng Ren, Philippe Franck, Min Tang, Aleksander Jaworski, Giovanni Barcaro, Susanna Monti, Lata Chouhan, Jabor Rabeah, Alina Skorynina, Joaquin Silvestre-Albero, Laura Simonelli, Anna Rokicińska, Elke Debroye, Piotr Kuśtrowski, Sara Bals, Shoubhik Das

**Affiliations:** a Department of Chemistry, University of Antwerp Antwerp Belgium Shoubhik.Das@uni-bayreuth.de; b Department of Chemistry, University of Bayreuth Bayreuth Germany; c EMAT and NANO Lab Center of Excellence, Department of Physics, University of Antwerp Antwerp Belgium; d Department of Materials and Environmental Chemistry, Stockholm University Stockholm Sweden; e CNR-IPCF, Institute for Chemical and Physical Processes via G. Moruzzi 1 56124 Pisa Italy; f CNR-ICCOM, Institute of Chemistry of Organometallic Compounds via G. Moruzzi 1 56124 Pisa Italy; g Department of Chemistry, KU Leuven Leuven Belgium; h Leibniz-Institut für Katalyse e. V Albert-Einstein-Straße 29a 18059 Rostock Germany; i CLAESS Beamline, ALBA Synchroton Spain; j Departamento de Quimica Inorganica-Instituto Universitario de Materiales, Universidad de Alicante Alicante E-03080 Spain; k Faculty of Chemistry, Jagiellonian University Krakow Poland

## Abstract

The transformation of CO_2_ into value-added products from an impure CO_2_ stream, such as flue gas or exhaust gas, directly contributes to the principle of carbon capture and utilization (CCU). Thus, we have developed a robust iron-based heterogeneous photocatalyst that can convert the exhaust gas from the car into CO with an exceptional production rate of 145 μmol g^−1^ h^−1^. We characterized this photocatalyst by PXRD, XPS, ssNMR, EXAFS, XANES, HR-TEM, and further provided mechanistic experiments, and multi-scale/level computational studies. We have reached a clear understanding of its properties and performance that indicates that this highly robust photocatalyst could be used to design an efficient visible-light-mediated reduction strategy for the transformation of impure CO_2_ streams into value-added products.

## Abstract 2

The transformation of CO_2_ into value-added products from an impure CO_2_ stream, such as flue gas or exhaust gas, directly contributes to the principle of carbon capture and utilization (CCU).

## Graphical abstract

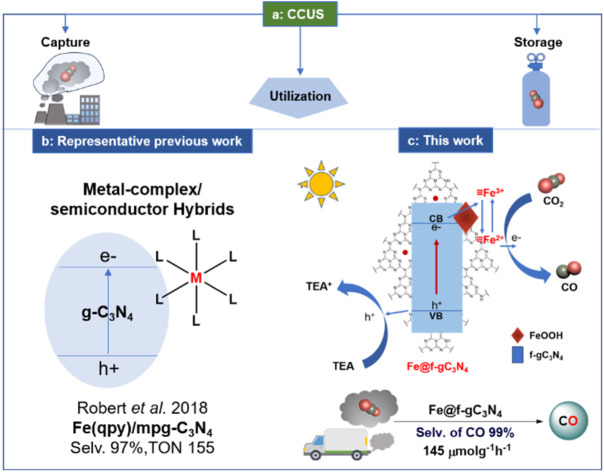

## Introduction

**Scheme 1:**
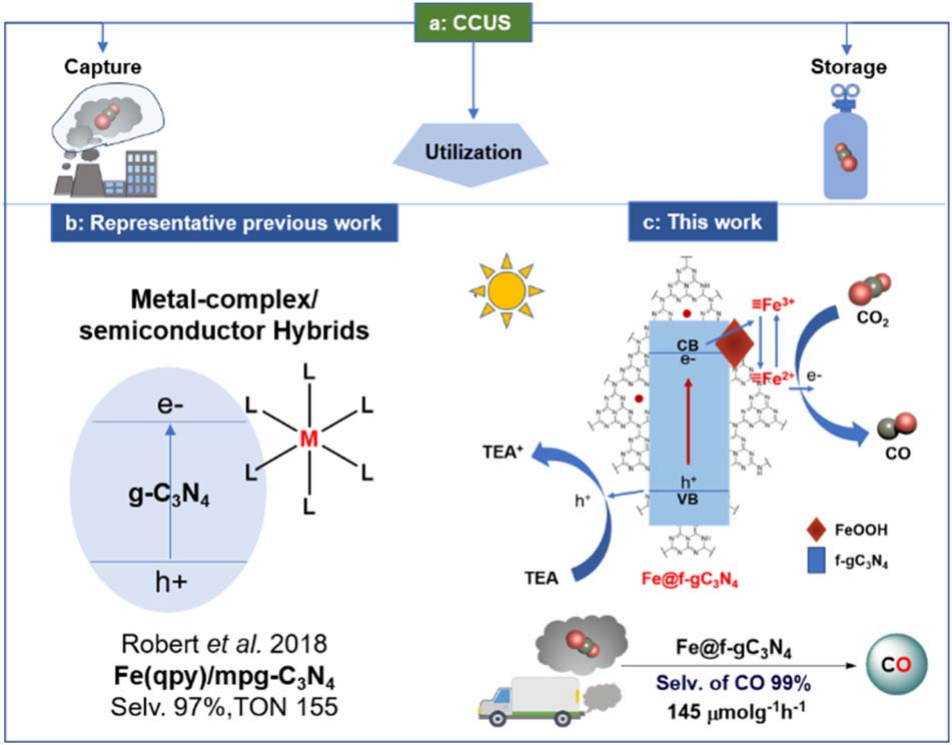
Reported photocatalytic approaches and our work for the reduction of carbon dioxide.

The development of iron-based catalysts is attractive due to the high abundance of iron in the Earth's crust and low cost compared to the other transition metals.^1–8^ Furthermore, iron can adopt diverse oxidation states (from −2 to +5) and can promote single electron transfer reactions.^9^ These advantages triggered scientists to develop novel iron-based homo-/heterogeneous catalysts, and many of them are comparable to those of the 4d and 5d-based transition metal analogs.^10–13^ Parallel to the development of iron-based catalysts, the direct transformation of CO_2_ into value-added products has tremendous potential because CO_2_ is non-toxic and abundant in the atmosphere.^14–27^ However, in most cases, only pure CO_2_ streams are used as carbon sources. If impure CO_2_ streams such as flue gas from industries or exhaust gas from a car could be utilized, it will avoid the associated cost and energy requirement for the CO_2_ purification procedure. This new approach contributes to the Carbon Capture and Utilization (CCU) principle (Scheme 1a).^28–39^ Nonetheless, the presence of impurities such as O_2_, water vapor, CO, NO_*x*_, and hydrocarbons in the impure CO_2_ stream can be harmful to the photocatalyst and can be detrimental to the desired product formation.^40–44^

Recently, graphitic carbon nitrides (g-C_3_N_4_) have become highly attractive as photocatalysts due to their high chemical and thermal stability, appropriate band structures, and low cost.^45–51^ Thus, researchers have employed g–C_3_N_4_–based hybrid photocatalysts (g-C_3_N_4_ combined with Ru,^52^ Co,^53–56^ and Fe^57–59^-based metal complexes) to improve the selectivity and reactivity for the reduction of CO_2_ into CO (Scheme 1b). Among them, Co-based transition metal complexes such as [Co(bpy)_3_]^2+^ and Co(qpy) along with g-C_3_N_4_, exhibited the selectivity of 86 and 98% (TON of CO were 3.7 and 128, with the production rate of 37 and 7.98 μmol g^−1^ h^−1^ respectively) for the formation of CO in the presence of triethanolamine (TEOA) and 1,3-dimethyl-2-phenyl-2,3-dihydro-1*H*-benzo[*d*]imidazole (BIH) as sacrificial reductant.^54,56^ Additionally, Fe-based metal complexes such as Fe(qpy)_3_ and Fe(qpy)-BA exhibited the formation of CO with a production rate of 91 and 141 μmol g^−1^ h^−1^ respectively (selectivity was 97 and 95% in the presence of TEOA and TEA + BIH as sacrificial reductant).^57,58^ However, these hybrid systems always required expensive ligands and multistep synthetic routes (associated with the metal complexes), exhibited poor recyclability, and rarely showed reactivity toward the transformation of an impure CO_2_ stream.

To achieve a robust and recyclable Fe-based photocatalyst, construction of composite material could be a promising route since this could address the rapid recombination of photogenerated carriers which are the common limitations for the photocatalytic reactivity of g-C_3_N_4_.^60,61^ Along this direction, amorphous iron-based oxyhydroxides (FeOOH), known as promising Fenton-like catalysts, should be ideal for hybridization due to their small particle size.^62–64^ The coupling of amorphous FeOOH with g-C_3_N_4_ could be a sound strategy for constructing an effective recyclable photocatalyst. Considering this, we first modified the g-C_3_N_4_ moiety by introducing an aryl functionality (f-gC_3_N_4_) to improve the photocatalytic properties of g-C_3_N_4_ (ref. 49) and later, FeOOH was introduced into the moiety to fabricate a FeOOH/f-gC_3_N_4_ composite photocatalyst. This photocatalyst generated CO with a production rate of 304 μmol g^−1^ h^−1^ with a selectivity of 99%. Expediently, when exhaust gas from a car was applied, CO was formed with a production rate of 145 μmol g^−1^ h^−1^ (Scheme 1c), thanks to the enhanced interfacial electron transfer between FeOOH and f-gC_3_N_4_. To the best of our knowledge, this is the first Fe-based recyclable photocatalytic system that can be applied for the reduction of impure CO_2_ stream.

## Results and discussion

At the beginning of this project, g-C_3_N_4_ was synthesized by calcinating dicyandiamide (DCDA) at 550 °C for 4 h (temp. increasing rate = 2.2 °C min^−1^) in a tube furnace under aerobic conditions (please see the detailed procedure in the ESI†).^49^ Followed by this method, functionalized graphitic carbon nitrides (f-gC_3_N_4_) were achieved by stirring a mixture of 9 gm of DCDA and 150 mg of 2-amino-5-trifluoromethyl benzonitrile in deionized water (45 mL) at 95 °C until it’s completely dried, resulting mixture was then grinded in an algae mortar and was calcined at 550 °C for 4 h (temp. increasing rate = 2.2 °C min^−1^) under aerobic conditions (please see in the ESI†). Later, Fe(NO_3_)_3_·9H_2_O (7.23 mg for 0.2Fe@f-gC_3_N_4,_ 18 mg for 0.5Fe@f-gC_3_N_4_, 25.3 mg for 0.7Fe@f-gC_3_N_4_, and 36 mg for 1Fe@f-gC_3_N_4_) and f-gC_3_N_4_ (500 mg) were mixed in 10 mL deionized water and the reaction mixture was further stirred at 100 °C (please see in the ESI† for the detailed procedure). It should be noted that 0.2Fe@f-gC_3_N_4_, 0.5Fe@f-gC_3_N_4_, 0.7Fe@f-gC_3_N_4_ and 1Fe@f-gC_3_N_4_ denotes 0.2, 0.5, 0.7 and 1 wt% of iron loading on f-gC_3_N_4_ respectively. After the synthesis of all these photocatalysts, the Tauc plot exhibited that gC_3_N_4_, f-gC_3_N_4_, 0.5Fe@g-C_3_N_4,_ 1Fe@f-gC_3_N_4_, 0.7Fe@f-gC_3_N_4_, 0.5Fe@f-gC_3_N_4_, and 0.2Fe@f-gC_3_N_4_ had the band gap of 2.64, 2.48, 2.46, 2.34, 2.47, 2.46 and 2.46 eV, respectively (Fig. S3†). Furthermore, Mott–Schottky plots of all these photocatalysts disclosed that the flat band (fb) potential of gC_3_N_4_, f-gC_3_N_4_, 0.5Fe@g-C_3_N_4_, 1Fe@f-gC_3_N_4_, 0.7Fe@f-gC_3_N_4_, 0.5Fe@f-gC_3_N_4_, and 0.2Fe@f-gC_3_N_4_ were −0.34, −0.44, −0.47, −0.52, −0.56, −0.33 and −0.38 eV *vs.* Normal Hydrogen Electrode (NHE). Additionally, the positive slope indicated the n-type nature of these semiconductors (Fig. S4†). The conduction band (CB) of an n-type inorganic semiconductor is commonly assumed to be ≈−0.2 V negative than the flat band potentials. Thus, the CB potentials were derived by lowering the flat band potential by 0.2 V compared to the NHE (Fig. S5†).^47,65^

**Fig. 1:**
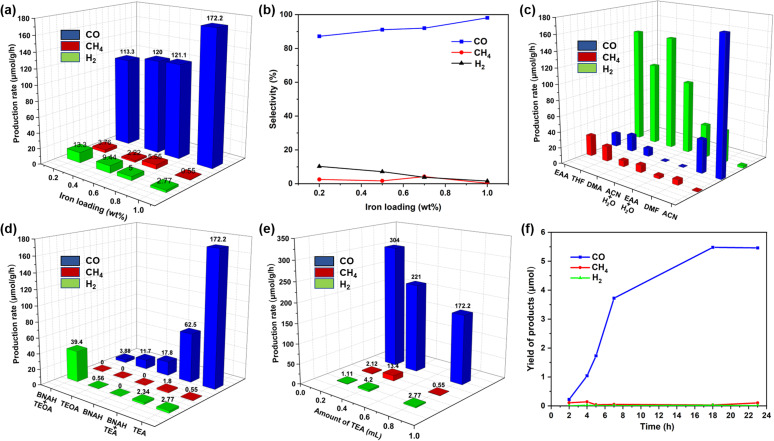
(a) Reduction of CO_2_ by using different loading of iron catalysts; (b) selectivity in CO_2_ reduced products by using different loading of iron catalysts; (a and b) reaction conditions: iron catalyst (1 mg), TEA (0.8 mL), ACN (3.2 mL), time = 18 h, *λ* = 427 nm, reaction temperature = 30 °C. (c) Photocatalytic CO_2_ reduction by using different solvents. Reaction conditions: 1Fe@f-gC_3_N_4_ (1 mg), TEA (0.8 mL), different solvents (3.2 mL), time = 18 h, *λ* = 427 nm, reaction temperature = 30 °C. (d) Photocatalytic reduction of CO_2_ by using different reductants. Reaction conditions: 1Fe@f-gC_3_N_4_ (1 mg), different reductant for single reductant system TEA or TEOA (0.8 mL), 0.1 M BNAH, for binary reductant system BNAH (32 mg) + TEOA (0.4 mL), BNAH (32 mg) + TEA (0.4 mL), ACN (3.2 mL), time = 18 h, *λ* = 427 nm, reaction temperature = 30° (e) production rate of photocatalytic CO_2_ reduction by using different amount of TEA. Reaction conditions: catalyst 1Fe@f-gC_3_N_4_ (1 mg), TEA (0.2, 0.4, 0.8 mL), solvent ACN (3.2–3.8 mL), time = 18 h, *λ* = 427 nm, reaction temperature = 30 °C. (f) Kinetic studies for the photocatalytic reduction of pure CO_2_. Reaction conditions: 1Fe@f-gC_3_N_4_ (1 mg), TEA (0.2 mL), ACN (3.8 mL), time = 2–23 h, *λ* = 427 nm, reaction temperature = 30 °C.

After the synthesis, photocatalytic experiments were carried out in 4 mL of CO_2_-saturated acetonitrile solution in the presence of a freshly distilled sacrificial electron donor (ACN : triethylamine, 4 : 1 V/V), under the irradiation of a Kessil lamp for 18 h (*λ* = 427 nm, 100 mW cm^−2^ light intensity, Table S1†). Indeed, CO was the primary product, with a minor quantity of CH_4_ and H_2_ In this photocatalytic reaction, TEA got oxidized to form TEAH^+^.^58^ To our observation, only a trace amount of CO and H_2_ were obtained in the case of g-C_3_N_4_, while a moderate amount of CO with 91% selectivity was observed in the presence f-gC_3_N_4_. Furthermore, when 0.5 wt% of Fe was deposited onto both gC_3_N_4_ and f-gC_3_N_4_, the Fe@f-gC_3_N_4_ system exhibited nearly 12 times higher production rate of CO. To investigate the superior role of 0.5 wt% Fe@f-gC_3_N_4_ photocatalyst, pure Fe(NO_3_)_3_·9H_2_O was mixed externally with f-gC_3_N_4_ (iron content was the same as 0.5Fe@f-gC_3_N_4_). A lower quantity of CO clearly confirmed the importance of the deposition of iron onto f-gC_3_N_4_ structure. It could be due to the fact that the metal deposition enhanced the charge transfer efficiency from the conduction band of f-gC_3_N_4_ to the active metal site of Fe^+*n*^ and that was ideal for the effective reduction of CO_2_.^57^ After that, we were able to further increase the catalytic reactivity through different loadings of iron (0.2–1 wt%) on f-gC_3_N_4_ (Fig. 1a and b). We observed that the production rate of CO was linearly increased up to 0.7 wt% and was drastically boosted for 1Fe@f-gC_3_N_4_ (172 μmol g^−1^ h^−1^). Indeed, iron sites are prone to adsorb CO_2_; therefore, with the increase of iron loadings, more electrons will be transferred to the iron sites to reduce CO_2_. Thus, the increased Fe-loading prolonged the lifetime of the charge carriers and enhanced the transfer of the photogenerated electrons from f-gC_3_N_4_ to the Fe^+*n*^ center and then to CO_2_. Instead, a smaller number of electrons were transferred toward proton reduction, which in turn suppressed H_2_ evolution,^67^ and the production rate and selectivity to CO were linearly increased with the increase of iron loading.

Then, we evaluated the importance of different solvents (CH_3_CN, DMF, DMA, EtOAc, THF and H_2_O) in the presence of 1Fe@f-gC_3_N_4_ (Fig. 1c). While all of them were favorable for this transformation, no reaction took place in pure water, and further addition of water to organic solvents such as CH_3_CN and EtOAc resulted in a lower evolution rate of CO. Surprisingly, in the aqueous binary solvent system, CH_4_ and H_2_ were the major products, and CO was the minor product (Fig. 1c). In fact, by adding 37% of water into CH_3_CN and EtOAc, the selectivity of the CO_2_ reduction product was changed entirely from CO to CH_4_ (8e^−^/8H^+^ reduction process) with a production rate of 9.30 and 3.53 μmol g^−1^ h^−1^ respectively. This could be attributed to the fact that the addition of water increased the number of available protons in the solution, which in turn took part in the CO_2_ reduction process to form CH_4_.^68^ Nevertheless, among all the solvents, CH_3_CN was the best for the photochemical reduction of CO_2_ to CO, with a high production rate of 172.2 μmol g^−1^ h^−1^ and an excellent selectivity of 98%. Additionally, sacrificial reductants such as triethylamine (TEA), triethanolamine (TEOA) and 1-benzyl-1,4-dihydronicotinamide (BNAH) were also investigated and a production rate of 172, 11.7, and 17.8 μmol g^−1^ h^−1^ with 98, 95, and 100% selectivity were obtained (Fig. 1d). Further investigations by using different amounts of TEA and reducing the amount of TEA to 0.2 mL resulted in an excellent production rate of 304 μmol g^−1^ h^−1^ (Fig. 1e). We argued that a higher concentration of TEA probably quenched the excited state of the photocatalyst and decreased the photocatalytic efficiency.^69^

Furthermore, control experiments suggested that in the absence of CO_2_, photocatalyst, and light, no formation of CO was observed (Fig. S8†). On the other hand, in the absence of TEA, the formation of CO was observed, but with a lower production rate of 76.6 μmol g^−1^ h^−1^. In these conditions, the kinetics of CO_2_ reduction exhibited a linear increase in CO production up to 18 h, and after this, the yield of CO remained constant but the production of CH_4_ was slightly increased. Furthermore, kinetic studies demonstrated that the evolution of CO was stable up to 23 h, which was comparable with the recently reported photocatalysts (Fig. 1f).^57,70^

**Fig. 2:**
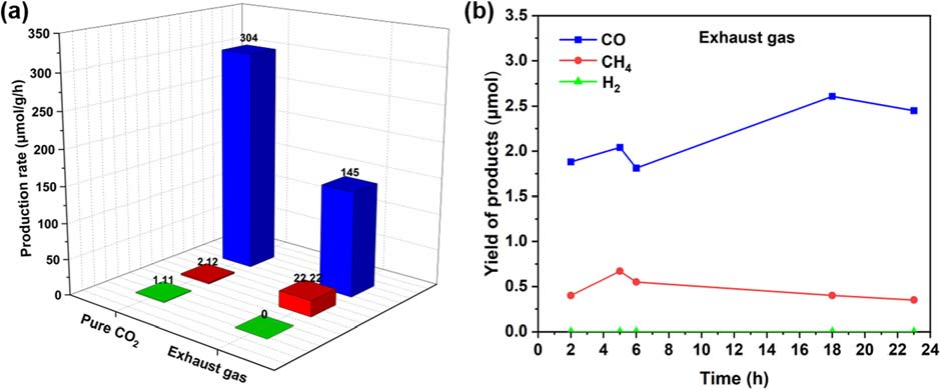
(a) Comparison of photocatalytic CO_2_ reduction by using pure CO_2_ and by using exhaust gas. Reaction conditions: 1Fe@f-gC_3_N_4_ (1 mg), TEA (0.2 mL), ACN (3.8 mL), time = 18 h, *λ* = 427 nm, reaction temp. = 30 °C. (b) Kinetic studies for the photocatalytic reduction of exhaust gas. Reaction conditions: 1Fe@f-gC_3_N_4_ (1 mg), TEA (0.2 mL), ACN (3.8 mL), *λ* = 427 nm, reaction temp. = 30 °C.

To demonstrate the application of this chemistry, the exhaust gas (containing impurities besides CO_2_, are shown in Fig. S7†) was directly collected from a vehicle by using gas sampling bags (Fig. S6†) and was applied directly under these reaction conditions, showing a CO production rate of 145 μmol g^−1^ h^−1^ (Fig. 2a). This decreased catalytic reactivity was due to the combined result of lower CO_2_ concentration in the exhaust gas as well as the presence of NO_*x*_ or SO_*x*_ in the exhaust gas, which can also be adsorbed on the active catalytic sites.^71^ In addition, the kinetic studies demonstrated the high stability of this photocatalyst in the presence of impurities such as H_2_, CO, CH_4_, O_2_, N_2_, and others, which are typically present in car exhaust gas (Fig. 2b). Furthermore, to estimate the reusability of this photocatalyst, 10 mg of the material was successfully used (under the same reaction conditions) and recycled for up to three cycles (Fig. S10†).

**Fig. 3:**
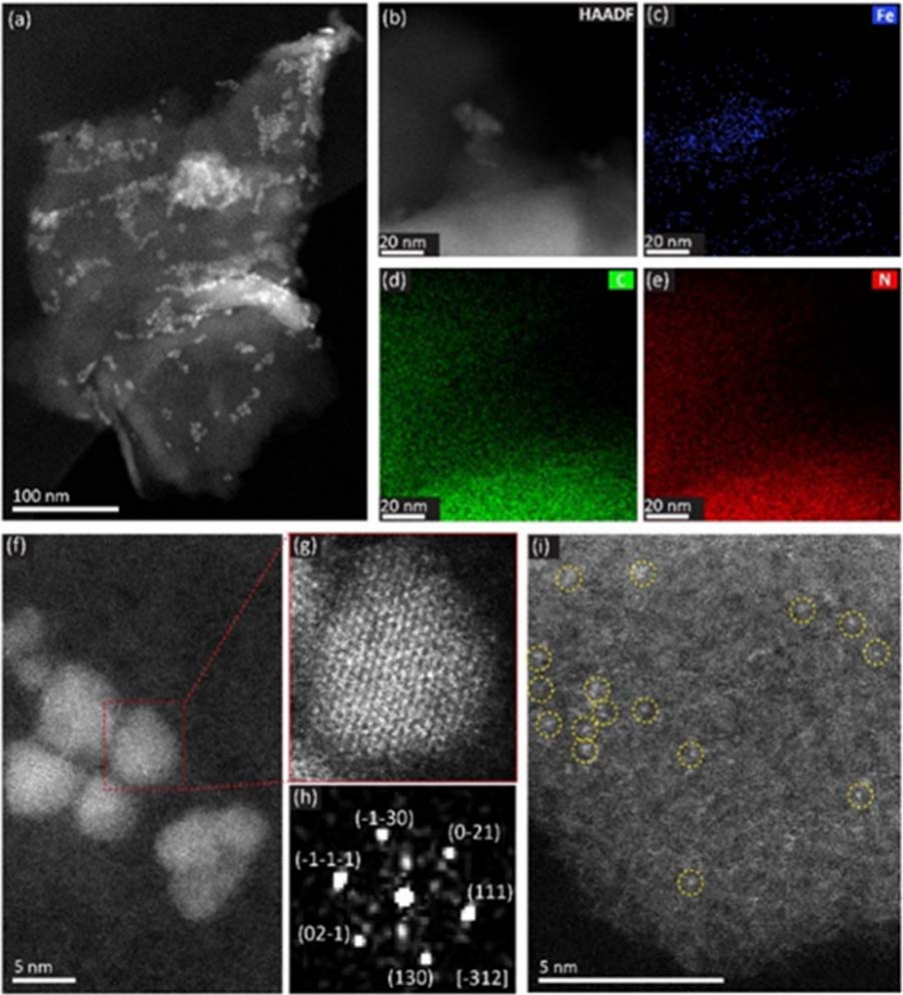
(a) HAADF-STEM image showing the morphology of 1Fe@f-gC_3_N_4_. (b–e) HAADF-STEM image (b) and EDX mapping results (c–e) showing the support f-gC_3_N_4_ and the Fe nanoparticles. (f) Atomic resolution HAADF-STEM image showing the Fe_2_O_3_ nanoparticles. (g) Image of magnified area in f marked by red dashed rectangles. (h) Fast Fourier transform (FFT) pattern of the FeO(OH) nanoparticles in 1Fe@f-gC_3_N_4_ (g). The direction is FeO(OH) [−312]. (i) Atomic resolution image showing the Fe clusters.

To achieve a deep characterization of the synthesized materials, we investigated the morphology and the structure of 1Fe@f-gC_3_N_4_ by High-Angle Annular Dark Field Scanning Transmission Electron Microscopy (HAADF-STEM). As shown in Fig. 3a, nanoparticles (NPs) were non-uniformly distributed over f-gC_3_N_4_, and EDX-mapping demonstrated that the NPs contained Fe (Fig. 3b–e). Atomic resolution HAADF-STEM images were acquired at lower (Fig. 3f) and higher (Fig. 3g) magnification. Fast Fourier transform (FFT) of the NPs marked by the red dashed rectangle in Fig. 3g provided lattice spacings of 2.690 Å, 2.580 Å, and 2.446 Å (Fig. 3h), which corresponded to FeO(OH) (130), (0−21), and (111), respectively. These results demonstrated that the supported NPs correspond to FeO(OH) species, and further investigating the edge of the f-gC_3_N_4_ support, bright dots marked by yellow dashed circles were observed, corresponding to small clusters containing Fe (Fig. 3i).

The crystal structure of prepared gC_3_N_4_, f-gC_3_N_4,_ and 1Fe@f-gC_3_N_4_ were further investigated *via* XRD. Two distinct diffraction peaks were observed at 12.9° and 27.3°, corresponding to the (100) and (002) crystal planes of g-C_3_N_4_, related to the in-plane repeating *s*-triazine structural moieties and interlayer stacking of the conjugated aromatic ring (Fig. S11†).^72–74^ While for f-gC_3_N_4_, the peak position and the peak intensity were almost similar with gC_3_N_4_, in the presence of Fe, the intensity of diffraction peak (002 in case of 1Fe@f-gC_3_N_4_) was slightly decreased as well as a marginal shift to higher diffraction angle was observed.^73,75^ However, no obvious diffraction peak of Fe_2_O_3_ or FeO(OH) phase were detected in the pattern of 1Fe@f-gC_3_N_4_, due to relatively low amount of the Fe species.^66,76,77^ Additionally, the weakening of the intensity of (002) peak suggested a decrease in crystallinity, with a consequent increase in the number of defects. These could trap a large number of carriers, with a resulting increase in charge separation and an improvement of the photocatalytic performance.^75^

**Fig. 4:**
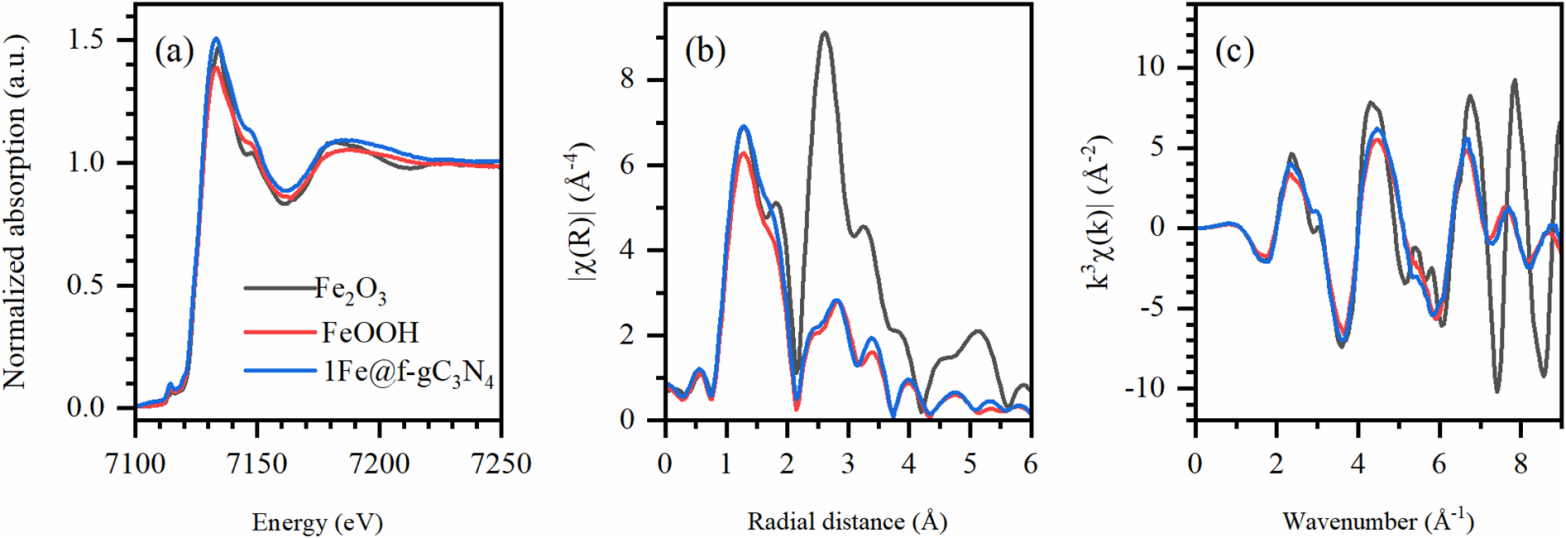
(a) Normalized XANES spectra, (b) FT-EXAFS spectra (phase uncorrected) and (c) *k*^3^-weighted EXAFS spectra at Fe K-edge for the 1Fe@f-gC_3_N_4_ and reference samples (Fe_2_O_3_ and FeOOH).

To verify the atomic environment of Fe in 1Fe@f-gC_3_N_4_, synchrotron X-ray absorption near-edge structure (XANES) and extended X-ray absorption fine structure (EXAFS) at Fe K-edge were performed and compared to the X-ray absorption spectra of the Fe_2_O_3_ and FeOOH reference materials. From the position of the rising edge in XANES spectra (Fig. 4a), it can be concluded that Fe in 1Fe@f-gC_3_N_4_ was predominantly in a +3 oxidation state, as confirmed by the close resemblance with Fe_2_O_3_ and FeOOH pre-edge peaks. EXAFS and Fourier transformed (FT) *k*^3^-weighted EXAFS curves were further extracted to probe the atomic iron-based local structures (Fig. 4b and c). When compared with the reference systems, 1Fe@f-gC_3_N_4_ nearly matched the peaks of FeOOH, which was especially noticeable for the shell scattering peaks of the longer-range order (2–3.5 Å), indicating Fe–Fe bonding. Concurrently, only FeOOH species could not fully describe XANES region and first shell, and a low Fe_2_O_3_ fraction below 5% cannot be excluded.

Solid-state MAS NMR spectra of all NMR-active nuclei present in the material (^1^H, ^13^C, ^15^N, and ^19^F) were also collected to probe the local structure and to examine potential structural changes upon doping of the f-gC_3_N_4_ with Fe^*n*+^ species (Fig. S12†). The ^1^H MAS, ^13^C CPMAS, and ^15^N CPMAS spectra were almost identical to NMR data we collected from the related polymeric carbon nitride catalysts and reported recently.^49,78^ Therefore, we concluded that the overall structure and polymerization degree in these materials were not affected to any significant extent by doping with Fe^*n*+^ ions. However, in the ^19^F MAS NMR spectrum, the ^19^F NMR shift of −120 ppm was distinct from that of −105 ppm observed by us for the undoped f-gC_3_N_4_, attributed to the presence of =CF_2_ groups (Fig. S12b†).^49,79,80^ Moreover, an additional weak signal at −202 ppm was detected, which had not been observed for the undoped material. The additional ^19^F MAS spectrum recorded using a short 0.2 s relaxation delay was collected to inspect if the appearance of the signal at −202 ppm could be related to the introduction of Fe^*n*+^ ions in the material. Upon using a short relaxation delay of 0.2 s, the intensity of the signal at −202 ppm was almost unaffected, whereas the signal at −120 ppm was significantly saturated. This could be attributed to the paramagnetic relaxation enhancement of the ^19^F nuclei in close contact with paramagnetic Fe^*n*+^ ions. The observed ^19^F NMR shifts of −120 and −202 ppm were most probably affected by the induced paramagnetic NMR shift interactions.^81^

The XPS spectra were recorded in the Fe 2p, C 1s, and N 1s regions for both fresh and used samples of 1Fe@f-gC_3_N_4_ (Fig. S13†). The dominant component of the pristine 1Fe@f-gC_3_N_4_ was the g-C_3_N_4_ phase, as confirmed by the C 1s and N 1s spectra. In the C 1s region, photoelectron emission, typical of sp^2^-bonded C atoms in N–C=N, was observed at 288.1 eV, while in the N 1s region three peaks at 398.6 eV (sp^2^-hybridized N atoms in C–N=C species), 400.1 eV (©dging N atoms N-(C)_3_ species) and 401.2 eV (N atoms in amino groups) were found.^82,83^ Fe^*n*+^ species also appeared on the surface of f-gC_3_N_4_. Due to the low content of this component, it was difficult to determine the chemical state of Fe unequivocally. Nevertheless, the Fe 2p_3/2_ peak position at 710.6 eV with an apparent multiplet splitting and a low satellite at *ca.* 718 eV indicated the presence of high-spin Fe^3+^ species. Another evidence confirming this supposition was the orbital splitting of Fe 2p_3/2_ and Fe 2p_1/2_ of 13.8 eV, which was similar to that reported previously in the literature for Fe^3+^.^84^ The elemental composition of the photocatalyst surface did not change during the process, and the used sample exhibited the same peaks in the individual XPS spectra. However, their intensity decreased due to the deposition of an additional component on the surface. Its nature was revealed by the XPS C 1s spectrum, where two new peaks were distinguished at binding energies of 284.8 eV and 285.9 eV, respectively. The former was typical for C–C/C=C, while the latter was for the oxidized C forms, most likely C–O or C=O.^85^

To obtain better insights into the mechanism of the reaction, the optical properties of the photocatalysts were characterized. Through UV/vis spectroscopy (Fig. S4a†), an absorption edge at 465 nm was observed in the case of gC_3_N_4_, whereas f-gC_3_N_4_, 0.5Fe@gC_3_N_4_ and 0.5Fe@f-gC_3_N_4_ exhibited a redshift of 30 nm, 34 nm and 34 nm, respectively (corresponding to 0.16 eV, 0.18 ev and 0.18 ev respectively). However, different Fe-loading had a negligible effect on the optical spectrum. The broad and intense absorption peak of the Fe-loaded f-gC_3_N_4_ catalyst in the visible region inferred that the catalyst could absorb more photons, which was a consequent enhancement of the photocatalytic activity.^86^ The difference in the absorption band between gC_3_N_4_ and Fe-loaded f-gC_3_N_4_ could be due to the electrostatic interaction between Fe^+*n*^ and f-gC_3_N_4,_ which promoted the electron delocalization throughout the heptazine framework by Fe^+*n*^.^87^ This was also evident from EPR spectroscopy, which showed a broadening of the 1Fe@f-gC_3_N_4_ signal (compared to f-gC_3_N_4_ one), due to the mutual interaction between the paramagnetic species (Fig. S14†).

The photocatalysts' steady-state photoluminescence (PL) spectra (Fig. S17a†) revealed emission maxima of gC_3_N_4_ at 471 nm and of Fe-gC_3_N_4_ at 466 nm. The emission spectra of f-gC_3_N_4_ and Fe-loaded f-gC_3_N_4_ were broad and centered around 513 nm. To investigate the charge carrier generation and dynamics, we measured the PL decays of the photocatalysts at their respective emission wavelength (Fig. S17b and c†). The PL decay profiles of all samples were recorded by time-correlated photon counting (TCSPC) spectroscopy and fitted using a tri-exponential decay equation. The average PL lifetimes and the fitting parameters are given in Table S3†. According to the lifetime fitting parameters, f-gC_3_N_4_ and Fe-loaded f-gC_3_N_4_ had longer *τ*_1_ and *τ*_2_ values compared to the gC_3_N_4_ and Fe-gC_3_N_4_ catalysts. Additionally, upon increasing Fe-loading in f-gC_3_N_4_, the carrier lifetimes exhibited an increasing trend of all *τ*_1_, *τ*_2_, and *τ*_3_ values. Although there is a slight difference in the average lifetime of the photocatalysts, the iron loading in f-gC_3_N_4_ increases the average lifetime, resulting in enhanced photocatalytic activity. This result supported the above-explained enhanced photocatalytic reactivity upon increasing the iron wt% due to a more efficient transfer of photogenerated electrons from f-gC_3_N_4_ to Fe^+*n*^. Following the increase of the charge carrier lifetimes, the generated electrons were available for a longer time for the photocatalysis reaction to efficiently take place. Specifically, in 1Fe@f-gC_3_N_4_, the *τ*_3_ value, reflecting the lifetime of the free charge carriers that can diffuse over longer distances, has significantly increased.

In addition, to investigate the separation efficiency of photogenerated electron–hole pairs, we recorded the photocatalyst's transient photocurrent responses under the irradiation of a Kessil lamp (*λ* = 427 nm). Among all of them, 1Fe@f-gC_3_N_4_ exhibited the quickest and highest photocurrent which remained stable up to 5 cycles which indicated a more efficient charge separation and faster electron transfer rate to trigger a superior photocatalytic reactivity of the catalyst (Fig. S18b†). Furthermore, *in situ* EPR spectroscopy showed that the relative number of the photoexcited electrons of f-gC_3_N_4_ was higher than 1Fe@f-gC_3_N_4_ due to the facile electron transfer to Fe (Fig. S14†). Additionally, Electrochemical Impedance Spectroscopy (EIS) of gC_3_N_4_, f-gC_3_N_4_ and all the Fe-loaded gC_3_N_4_ and f-gC_3_N_4_ exhibited decreasing charge transfer resistance, demonstrating the presence of covalent linking in 1Fe@f-gC_3_N_4_ which significantly enhanced the conductivity (Fig. S18a†). The CO_2_ reduction was confirmed by a spin-trapping experiment with DMPO (Fig. S16†). The holes in the valence band were quenched by the sacrificial reductant (TEA) and decreased the electron–hole recombination, as it is evident from the *in situ* EPR investigations (Fig. S15†). In the valence band, TEA reacted with holes and subsequently formed the α-amino radical and protons.^88^

Further to gather detailed information about the reaction mechanism, computational calculations were done (see in the ESI†) and after ≈50 ps of RMD, the sampled structures showed that the adsorption tendency of CO_2_ on the metal centers was mainly due to the coordination of one of its oxygens (Fe–O distance about 2.1 Å),^89^ and no reaction mechanisms were observed in those conditions. Indeed, the conversion started from an activated adsorption configuration where the carbon atom was connected to the metal center, and the CO_2_ molecule adopted a bent arrangement (CO_2_˙^−^).^90–92^ Experimentally, this activation was obtained by light irradiation (*λ* = 427 nm), which induced a charge transfer from the catalyst to the molecule, CO_2_ chemisorption with the elongation of the C–O bond, bending of O–C–O angle, and finally, dissociation of CO_2_ on the catalyst surface into CO and O species.^93,94^

We could mimic this process by including, in the RMD simulations, an electric field in the plane of the melem units (*x* direction, −0.01 V Å^−1^). The effect of the external electric field is a perturbation of the atomic charges of the system and, thus, a distortion of the molecular charge distribution. The evolution of the dipole moments imitated a charge transfer from the metal center to the adsorbed CO_2_. This is apparent in the atomic charge distribution plots of Fig. S21,† where it is evident the charge transfer from Fe to CO_2_ during the first 7.5 ps of the simulation (Fig. S21† – bottom) and the change of nature of the C atom when CO_2_ reduces to CO (Fig. S21† – middle plot). The adsorbed CO_2_ in a bent geometry was negatively charged. Indeed, from the beginning of the polarized dynamics, CO_2_ changed from an extended to a bent conformation and remained adsorbed on the Fe atom through its carbon (Fig. S20†). The stabilized complex received a proton from the solution and formed the adsorbed *COOH species. This species was short-lived, and the OH was quickly released in solution and then protonated. In contrast, CO remained stably adsorbed on the metal center (Fig. S22†). Besides reproducing possible hydrogen exchanges between the solvent and the region around the COO–Fe complex, the mechanisms produced a water molecule that freely migrated in the solution, whereas CO remained connected to the metal.

**Fig. 5:**
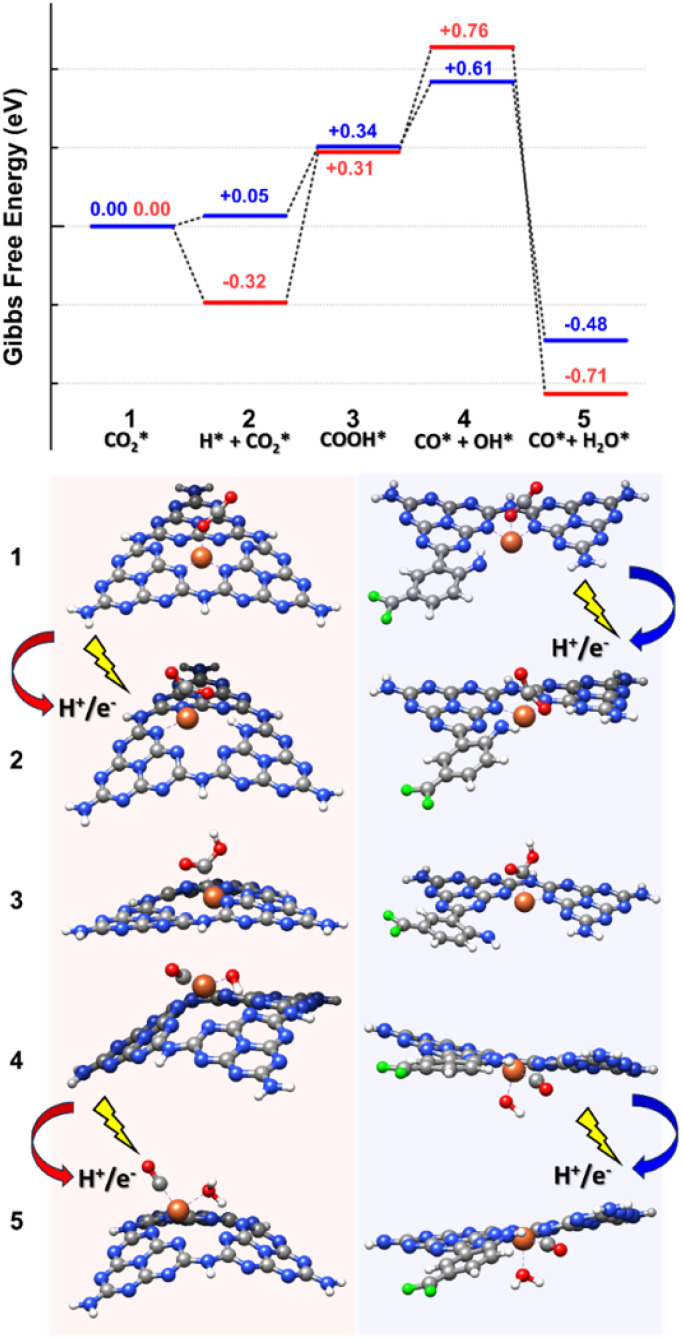
Reaction mechanism showing CO_2_ reduction to CO involving Fe atom in a high-coordination (red path) and a low-coordination (blue path) sites. State 1 is chosen as a reference for energy estimation, whereas the free energies of the other states (from 2 to 5) are estimated at the DFT level by employing the Norskov model.^96^ Color code: C gray, N blue, F green, Fe orange, O red, H white.

**Fig. 6:**
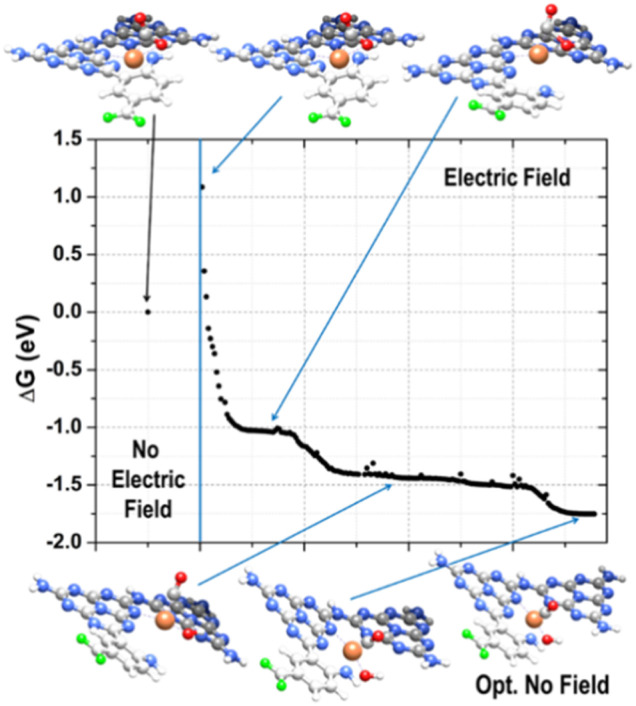
Reaction path of the adsorbed CO_2_ reduction to CO. The DFT minimum energy structures (initial and final points – top left and bottom right ball and stick models) are shown together with the intermediate species indicated by the blue arrows. Color code: C gray, N blue, F green, Fe orange, O red, H white.

To refine this picture, we extracted the MD snapshots describing the primary steps of the reaction mechanism, size-reduced them to three melem units with a chelated Fe atom and an adsorbed CO_2_ molecule (high-coordination site) or two melem units with a chelated Fe-atom, the f-substituent, and an adsorbed CO_2_ molecule (low-coordination site), and carried out density functional theory (DFT) calculations with Gaussian16.^95^ These were used to estimate minimum energy structures, charge analyses, possible reaction paths, and relative energy barriers (Fig. 5, 6 and S23†). We optimized the geometries in ACN through the integral equation formalism variant (IEFPCM) of the Polarizable Continuum Model (PCM), using the B3LYP-D3(BJ) functional with the Grimme D3 correction (Becke–Johnson parameters^97^), to account for the van der Waals interactions, and the 6-31(d,p) basis set for all the elements except Fe, which was described with the def2-TZVP basis set.^98^ Charge analysis was performed using NBO (full Natural Bond Orbital).^99,100^

Both high- and low-coordination sites provide a similar qualitative picture of the reaction mechanism, in agreement with the results of RMD simulations. In the absence of external stimuli, 
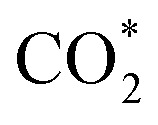
 molecule interacted weakly *via* one of its oxygens with the catalyst metal center (Fe–O equilibrium distance is 2.04 Å, in fair agreement with the results of RMD), which acted as a Lewis acceptor (see state 1 in Fig. 5), as confirmed by the small positive charge carried by CO_2_ (about +0.2*e*) and the charge decrease on Fe after adsorption (about 1.1*e*, to be compared with 1.3*e* of a naked Fe atom – Fig. S23†). The 
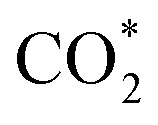
 activation happened after the addition of a H^+^/e^−^ couple promoted by UV irradiation: a similar bent adsorption configuration obtained by RMD (and shown in state 2 of Fig. S23†) was stabilized by a flow of negative charge to both the CO_2_ molecule (charge of ≈ –0.4*e*) and the Fe center (charge decrease from ≈+1.0*e* to ≈+0.7*e*). The proton (as in RMDs) was adsorbed on a nitrogen atom at the edge of a neighboring melem unit. CO_2_ negative charging is also evident in the PDOS shown in Fig. S24.†

After proton migration to 
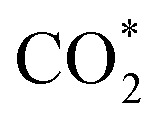
 we obtained the COOH* configuration shown in state 3 (Fig. 5). This positive charge flow allows negative charge back-migration from Fe to the adsorbate (with COOH almost neutral), as shown in Fig. S23.† The elongation and weakening of the bond between the carbon and the hydroxyl group (induced by the catalyst-adsorbate charge transfer, populating the p* LUMO of CO_2_ (ref. 88)) can be exasperated until the breaking of the C–O bond realized the configuration shown in state 4 (Fig. 5), which presents CO and OH separately adsorbed on the Fe atom. The positive charge carried by CO was compensated by a reduced charge on Fe (relative to state 2), and the negative charge carried by OH* (about −0.2*e*). States 3 and 4 are at higher energy relative to state 2 (≈1.08 eV for the high-coordination site and 0.56 eV for the low-coordination). The reduced energy difference characterizing the low-coordination site suggests that this can promote CO_2_ reduction easier than the high-coordination site and will be further investigated in the following. The UV-vis spectra simulated for state 2 of the low-coordination site, calculated at the TD-DFT level (Fig. S25†), show a dominant peak at about 465 nm, corresponding to a charge transfer from the metal ion to the adsorbed 
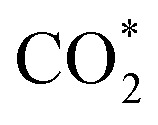
 molecule, aiding the C–O bond breaking in overcoming the observed energy difference between states 2 and 4. This agrees with the experimental choice of a blue LED (*λ* = 427 nm) as the excitation source.

Further addition of a H^+^/e^−^ couple (promoted by UV excitation) led to the configuration of state 5 (Fig. 5), which corresponded to adsorbed CO and H_2_O on the Fe catalyst. H_2_O carried a slight positive charge (about +0.2*e*), and CO kept its positive charge of about +0.4*e*. The relatively strong dipole between Fe and CO is a clear signal of a strong CO anchoring to the catalytic site, which can be disrupted if the charge flows from Fe to CO. The Gibbs free energy of this last state is lower than that of any other state investigated, confirming the thermodynamic tendency of this system to promote CO_2_ reduction. This sequence of events is the same as observed in RMD. We resorted to the electric field option already used for the RMD to recreate a possible dynamics mechanism at the DFT level instead of performing computationally expensive excited-state quantum chemistry simulations. Starting from the optimized complex of the low-coordination corresponding to state 2 (Fig. 5), where the CO_2_ molecule was adsorbed with the carbon atom on the metal ion, we emulated the first few steps of the reduction mechanism by applying an external electric field in the -x direction with a magnitude of 0.008 au.

From the examination of the free energy difference plot, it is evident that a low activation energy of ≈1.1 eV was necessary to start the process; this activation energy is reasonable when compared to the energy difference of ≈0.6 eV between states 2 and 4, estimated at the static level (Fig. 6). Then, the mechanism proceeded barrierlessly, passing through two intermediate metastable geometries (plateau regions). The first one is characterized by the appearance of the adsorbed COOH* species (state 3 in Fig. 5), whereas in the second one, the OH detached from C and became connected to the metal center (at a C–Fe–OH angle of about 103°, state 4 in Fig. 5), forming a bond with Fe (with an average Fe–O length of approximately 1.8 Å) and a hydrogen bond with the nitrogen atom of the nearby triazine ring (with an average OH–N distance of roughly 1.8 Å and a donor-H-acceptor angle of about 140°). In the final stable configuration, the hydrogen bond was lost, and the OH group moved to a farther N–O separation of about 3 Å but remained connected to the metal center. Interestingly, when simulated under the effect of the electric field, the energies of states 3 and 4 become lower than that of state 2, indicating a stabilizing effect played by the external perturbation on configurations which, when analyzed in their ground state, resulted instead higher in energy.

## Conclusions

In conclusion, we have successfully demonstrated that coupling amorphous FeOOH with f-gC_3_N_4_ exhibited an efficient visible-light-mediated CO_2_ conversion into CO. The presence of FeOOH in functionalized gC_3_N_4_ modulated the electronic interaction between Fe species and semiconductor, making an efficient photocatalyst for CO_2_ reduction. This FeOOH/f-gC_3_N_4_ composite heterogeneous photocatalyst has shown the highest CO evolution rate (so far reported among Fe-based heterogeneous photocatalysts) of 304 μmol g^−1^ h^−1^ with an excellent selectivity of >99%. This earth-abundant and low-cost photocatalyst also exhibited excellent reactivity and stability for reducing car exhaust gas, which clearly depicted the strong application potential of this chemistry. We strongly believe that our photocatalytic system will open a new strategy in the field of photocatalytic CO_2_ reduction.

## Author contributions

T. G., P. R. and S. D. conceptualized the project. S. D. supervised the project. T. G and P. R. synthesized the catalysts, conducted the catalytic experiments and the related data processing, and performed materials characterization and analysis with the help of P. F., A. J., A. R., P. K., L. C. and E. D. Furthermore, A. J. collected solid-state NMR spectra and J. R. performed EPR investigations. M. T. and S. B. conducted high-resolution, high angle annular dark-field transmission electron microscope (HAADF-STEM) spectroscopy. G. B. and S. M. performed the theoretical studies. J. S.-A., A. S. and L. S. performed X-ray absorption near edge structure (XANES) and extended X-ray absorption fine structure (EXAFS) measurement and analysis. A. C and E. D performed the steady state spectroscopy. A. R and P. K performed the X-ray photoelectron spectroscopy (XPS). The manuscript was written through the contributions of all authors.

## Conflicts of interest

There are no conflicts to declare.

## Supplementary Material

SC-015-D4SC02773F-s001
