# Supplementary material for: A robust Fe-based heterogeneous photocatalyst for the visible-light-mediated selective reduction of an impure CO2 stream
Source: Chem Sci. 2024 Jun 19;15(29):11488–99. doi: 10.1039/d4sc02773f (PMC11268485; doi:10.1039/d4sc02773f)
Supplement: SC-015-D4SC02773F-s001 [file SC-015-D4SC02773F-s001.pdf]

Electronic Supplementary Material (ESI) for Chemical Science.
This journal is © The Royal Society of Chemistry 2024

Supporting Information

A Robust Fe-Based Heterogeneous Photocatalyst for the Visible-Light-Mediated Selective Reduction of impure CO_2 Stream

Topi Ghosh[‡a], Peng Ren[‡a,b], Philippe Franck[a], Min Tang[c], Aleksander Jaworski[d], Giovanni Barcaro[e], Susanna Monti[f], Lata Chouhan[g], Jabor Rabeah[h], Alina Skorynina[i], Joaquin Silvestre-Albero[j], Laura Simonelli[i], Anna Rokicińska[k], Elke Debroye[g], Piotr Kuśtrowski[k], Sara Bals[c], and Shoubhik Das[a,b]*

[a]*Department of Chemistry, University of Antwerp, Antwerp, Belgium.*
[b]*Department of Chemistry, University of Bayreuth, Bayreuth, Germany*
[c]*EMAT and NANO lab Center of Excellence, Department of Physics, University of Antwerp, Antwerp, Belgium*
[d]*Department of Materials and Environmental Chemistry, Stockholm University, Stockholm, Sweden*
[e]*CNR-IPCF, Institute for Chemical and Physical Processes, via G. Moruzzi 1, 56124 Pisa, Italy*
[f]*CNR-ICCOM, Institute of Chemistry of Organometallic Compounds, s, via G. Moruzzi 1, 56124 Pisa, Italy*
[g]*Department of Chemistry, KU Leuven, Leuven, Belgium*
[h]*Leibniz-Institut für Katalyse e. V. Albert-Einstein-Straße 29a, 18059 Rostock, Germany*
[i]*CLAESS beamline, ALBA Synchroton, Spain.*
[j]*Departamento de Quimica Inorganica-Instituto Universitario de Materiales, Universidad de Alicante, Alicante E-03080, Spain*
[k]*Faculty of Chemistry, Jagiellonian University, Krakow, Poland*

‡ Denotes equal contributions

Corresponding authors: shoubhik.das@uni-bayreuth.de

S1. Experimental procedures

S1.1 Chemicals

Dry acetonitrile (99.9%), dicyandiamide (DCDA) and other reagent grade solvents were purchased from thermo-scientific. Iron (III) nitrate nonahydrate and triethanolamine (TEOA, ≥99%) and 1-benzyl 1,4-dihydronicotinamide (BNAH) were obtained from Sigma-Aldrich. Triethylamine (TEA, ≥99%) was obtained from Acros. All reagents were used without further purification except triethylamine. In catalytic reaction the triethylamine is used after purification.

S1.2 Preparation of catalyst

S1.2.1. Preparation of g-C_3N_4

9 g of DCDA was calcined at 550 °C for 4h (temp. increasing rate = 2.2°C/min) in a tube furnace under aerobic conditions. The sample was removed when the furnace temperature was cooled down to room temperature (24 °C). After calcination, around 3g of g-C_3N_4 catalyst was prepared and then was grinded to fine powder in an algae mortar.

S1.2.2 Preparation of f-gC_3N_4

A 100 mL beaker containing 9 g of DCDA, and 150 mg of 2-amino-5-trifluoromethyl benzonitrile and deionized water (45 mL) was stirred at 95 °C until it's completely dried. Resulting mixture was then grinded in an algae mortar and was calcined at 550 °C for 4h (temp increasing rate = 2.2°C/min) under aerobic conditions. The sample was removed when the furnace temperature was cooled down to room temperature (24 °C). After the calcination, the around 3g prepared material was grinded in an algae mortar.[1]

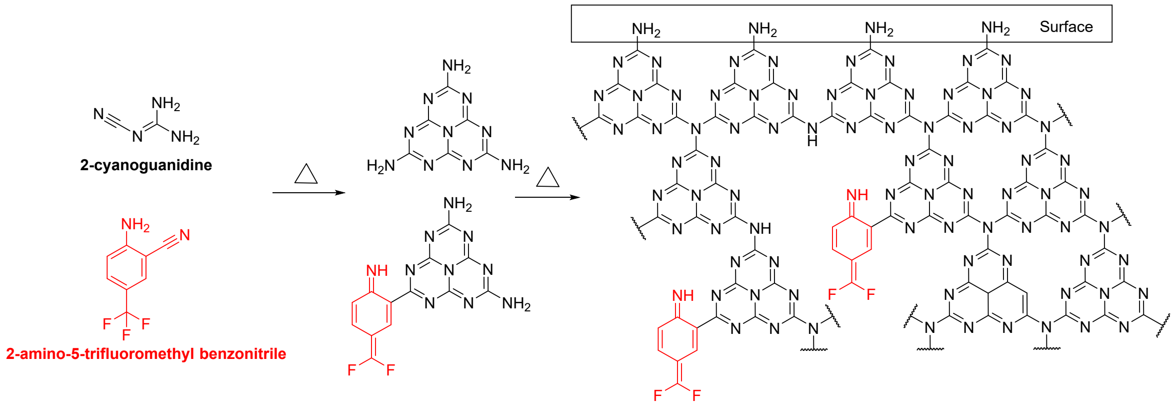

Scheme S 1. The schematic of preparation procedure of f-gC_3N_4 and the functionalization introduced in f-gC_3N_4 samples.

S1.2.3 Preparation of Fe@f-gC_3N_4 (0.2Fe@f-gC_3N_4, 0.5Fe@f-gC_3N_4, 0.7Fe@f-gC_3N_4 and 1Fe@f-gC_3N_4)

In a 20 mL glass tube, 18 mg of $Fe(NO_3)_3 \cdot 9H_2O$ and 500 mg of f-gC_3N_4 were added in 10 mL deionized water and the reaction mixture was stirred at 100°C until it's completely dried. Resulting mixture was then calcined in a muffle furnace at 300 °C for 2 h in the presence of N_2 (temp increasing rate = 2.2°C/min). The sample was removed when the furnace temperature was cooled down to room temperature (24 °C). After calcination the prepared

material was grinded in an algae mortar. 0.2Fe@f-gC_3N_4, 0.7Fe@f-gC_3N_4 and 1Fe@f-gC_3N_4 were prepared following the same procedure but by changing only the quantity of iron metal precursor ($Fe(NO_3)_3 \cdot 9H_2O$) such as 7.23 mg of $Fe(NO_3)_3 \cdot 9H_2O$ for 0.2Fe@f-gC_3N_4, 25.3 mg of $Fe(NO_3)_3 \cdot 9H_2O$ for 0.7Fe@f-gC_3N_4, 36 mg of $Fe(NO_3)_3 \cdot 9H_2O$ for 1Fe@f-gC_3N_4. The synthesis of 0.5Fe@gC_3N_4 followed the same procedure of 0.5Fe@f-gC_3N_4 by changing f-gC_3N_4 to gC_3N_4. 0.2Fe@f-gC_3N_4, 0.5Fe@f-gC_3N_4, 0.7Fe@f-gC_3N_4 and 1Fe@f-gC_3N_4 denoted 0.2, 0.5, 0.7 and 1 wt% of iron on f-gC_3N_4.

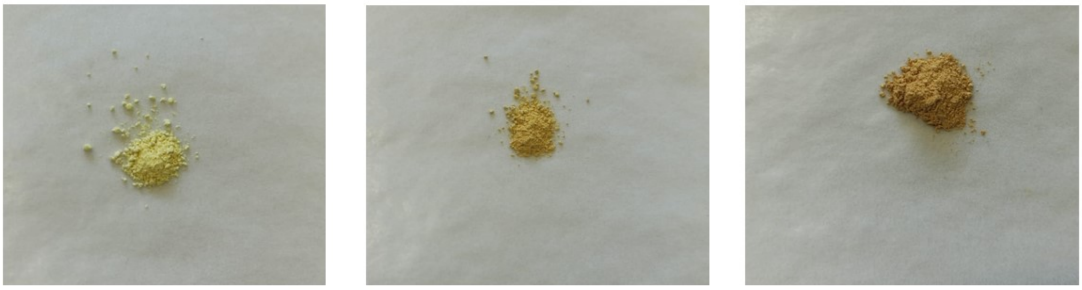

gC_3N_4 f-gC_3N_4 1Fe@f-gC_3N_4

Figure S 1. Photographs of the different catalysts after synthesis.

S1.3 Purification of triethylamine:

100 mL of triethylamine was stirred and distilled in the presence of 2.6 gm of calcium hydride. After the distillation, TEA was stored under nitrogen atmosphere in a dry schlenk flask over molecular sieves.

S1.4 Photocatalytic CO_2 reduction

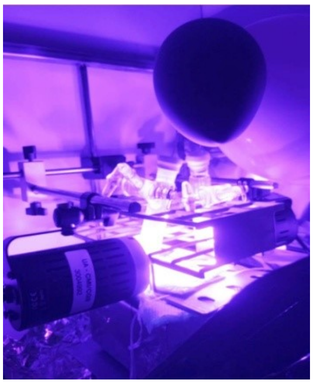

Figure S 2. Scheme of the experimental photocatalytic system. Photochemical reactor: gas–liquid system where the catalyst is heterogeneous (solid), the sacrificial reagent is homogeneous.

A 28 mL Schlenk tube containing 1 mg of the catalyst was capped with the rubber septum after the vacuum (3 minutes) and N_2 flow (30 seconds) to make anaerobic atmosphere inside the tube. This process was repeated for three times. After that, 0.2 mL of distilled TEA and 3.8 mL of dry ACN were added in the Schlenk tube. The solvent and TEA were added to the Schlenk tube in the presence of N_2 atmosphere. The reaction solution was then bubbled with CO_2 (99.9% pure) in the dark for 25 minutes. After CO_2 bubbling, a CO_2 balloon was kept on this Schlenk tube using a needle (needle above on the solution). The CO_2 saturated solution was then irradiated at λ = 427 nm kessil lamp (light intensity was 100 mW/cm^2 and distance between lamp to the Schlenk tube was ~1 cm, distance of fan from light set up was ~15 cm)

with vigorous stirring at 30 °C temperature for 18 h. The light set up containing two lights where each light was face to face parallel to each other and one Schlenk tube was placed in front of another Schlenk tube. After the reaction, gaseous products were measured by headspace GC machine (GC, Isbuan 345, model no: 1300, Global Analyser solution).

S2. Optimizations

S2.1 Tauc plots *via* UV-vis diffuse reflectance spectroscopy

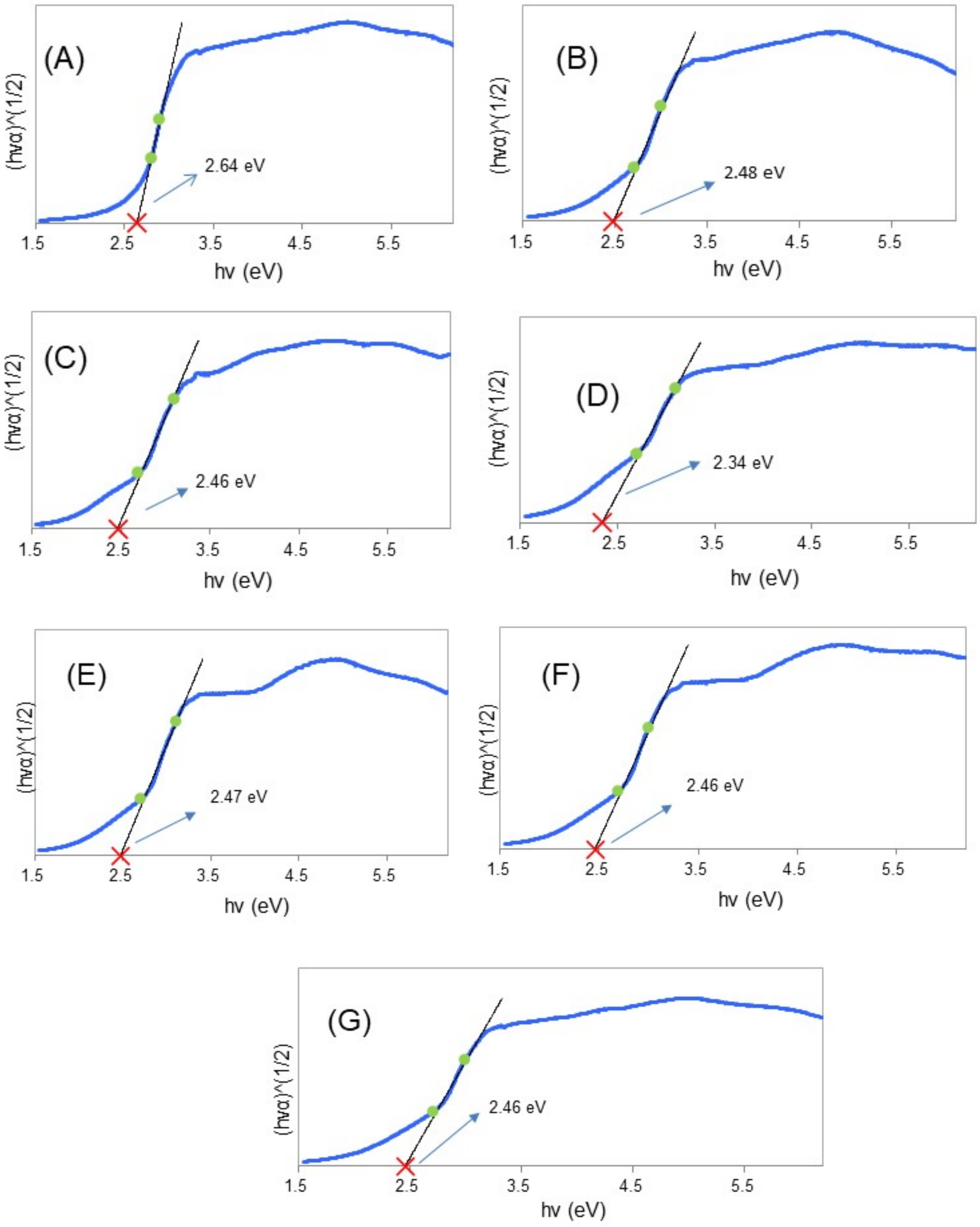

Figure S 3. Tauc plot of (a) gC_3N_4, (b) f- gC_3N_4, (c) 0.5Fe@ gC_3N_4, (d) 1Fe@f- gC_3N_4, (e) 0.7Fe@f- gC_3N_4, (f) 0.5Fe@f- gC_3N_4, (g) 0.2Fe@f-gC_3N_4.

The optical properties of samples were characterized by the UV-vis diffuse reflectance spectroscopy (Schimadzu UV-2600i, coupled with DRS integrating-sphere, where $BaSO_4$ was used as a reference standard). The photoelectrochemical measurements such as electrochemical impedance spectrum (EIS), Mott-Schottky tests and transient photocurrent

response were measured by using Metrohm Autolab potentiostat-galvanostat PGSTAT204. EIS was measured in the frequency range of 0.1-10^5 Hz. Transient photocurrent response curves were recorded under 40W kessil light (427 nm wavelength) conducted with no bias voltage with light on/off interval of 30 s in the presence of a 0.5 M sodium sulphate solution as electrolyte. All the above photoelectrochemical measurements were carried out by three electrode system such as catalyst was deposited on Fluorine Doped Tin oxide (FTO), Pt-foil and Ag/Agcl/3 (M) KCl electrode were used as working, counter and reference electrode, respectively. 5 mg of sample was added in a small vial containing ethanol (50 µL) and Nafion solution (5 wt%, 10 µL). The mixture was sonicated for one hour to make a homogeneous solution. After that, 15 µL of mixture was dispersed on the surface of FTO to obtain 1*1 cm^2 coating film. Furthermore, 0.5 M of sodium sulphate solution was degassed with nitrogen as electrolytic solution.

S2.2 UV-Vis DR spectra and Mott-Schottky diagram

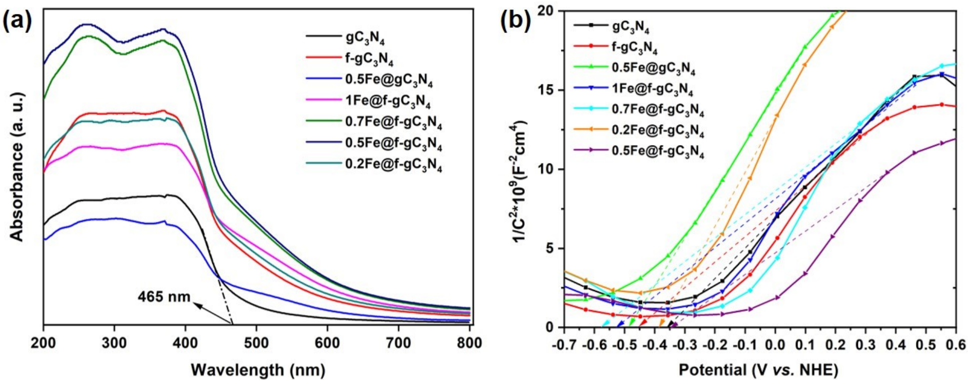

Figure S 4. (a): UV/Vis DR spectra, (b): Mott- Schottky diagram.

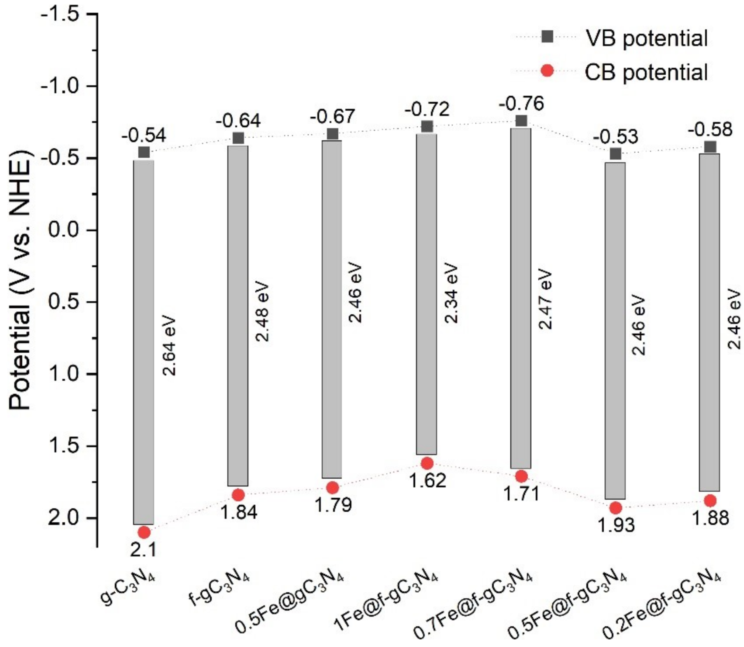

Figure S 5. Band position of different catalysts.

Table S1. Photocatalytic reduction of CO_2 by different catalysts.

Entry	Catalyst	Yield (μmol)			Selv. of CO (%)	TON (CO)
		CO	CH_4	H_2		
1	g-C_3N_4	0.01	-	0.03	25	-
2	f-gC_3N_4	1.30	0.08	0.05	91	-
3	0.5Fe@gC_3N_4	0.18	0.13	0.93	90	2.25
4	0.5Fe@f-gC_3N_4	2.15	0.04	0.17	91	26.87
5	$Fe(NO_3)_3.9H_2O$/gC_3N_4	0.03	0.006	0.33	8.3	0.37

Reaction conditions: Photocatalysts (entries 1-5), TEA (0.8 mL), ACN (3.2 mL), Time = 18 h, λ = 427 nm, Reaction temperature = 30˚C.

Table S 2 Photocatalytic CO_2 reduction at different wavelengths.

Entry	Wavelength (nm)	CO ($\mu molg^{-1}h^{-1}$)	CH_4 ($\mu molg^{-1}h^{-1}$)	H_2 ($\mu molg^{-1}h^{-1}$)	Selectivity of CO (%)
1	390	137.2	9.44	0.55	93
2	427	172.2	0.55	2.77	98
3	456	49.4	11.6	2.22	76

Reaction conditions: 1Fe@f-gC_3N_4 (1 mg), TEA (0.8 mL), ACN (3.2 mL), Time = 18 h, Wavelength of light (390 - 427 nm), Reaction temp = 30˚C.

S2.3 The collection procedure and composition of exhaust gases

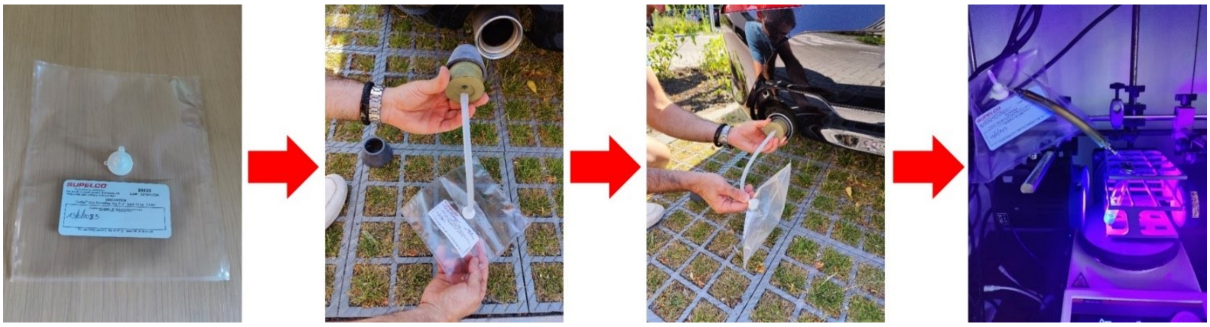

Figure S 6. The collection procedure of exhaust gasses.

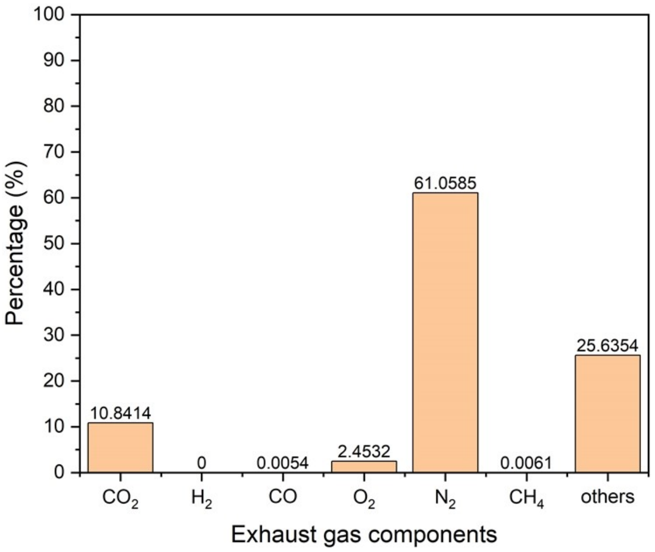

Figure S 7. The exhaust gas components.

The components of exhaust gas vary depending on the type of engine (e.g., gasoline, diesel) and the fuel used, but typically include a mixture of gases and particulate matter.

Others contains:

~12% water vapor.

~0.5% nitrogen oxides (NO_x, including NO and NO_2).

~0.05-0.5% hydrocarbons (HC, unburnt fuel).

Trace amounts of sulfur dioxide (SO_2)

Some amounts of particulate Matter (PM).

S2.4 Control experiments

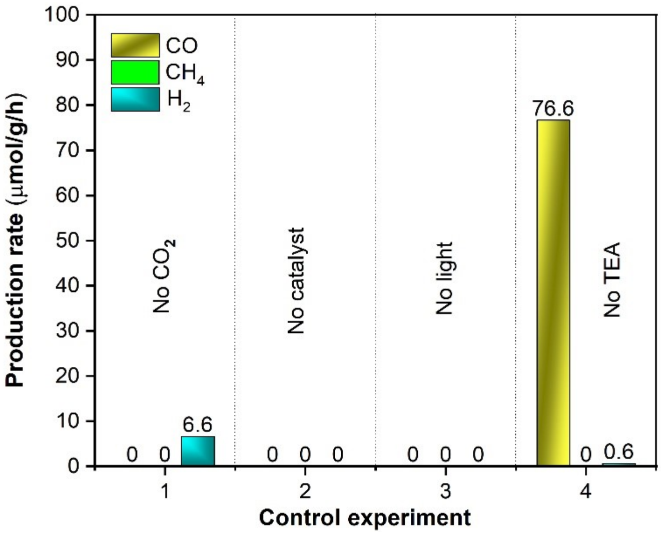

Figure S 8. Reaction conditions: 1Fe@f-gC_3N_4 (1 mg), TEA (0.2 mL), ACN (3.8 mL), Time = 18 h, Wavelength of light = 427 nm, Reaction temp = 30˚C.

S2.5 Photocatalytic CO_2 reduction by changing the amount of catalyst (1- 4 mg)

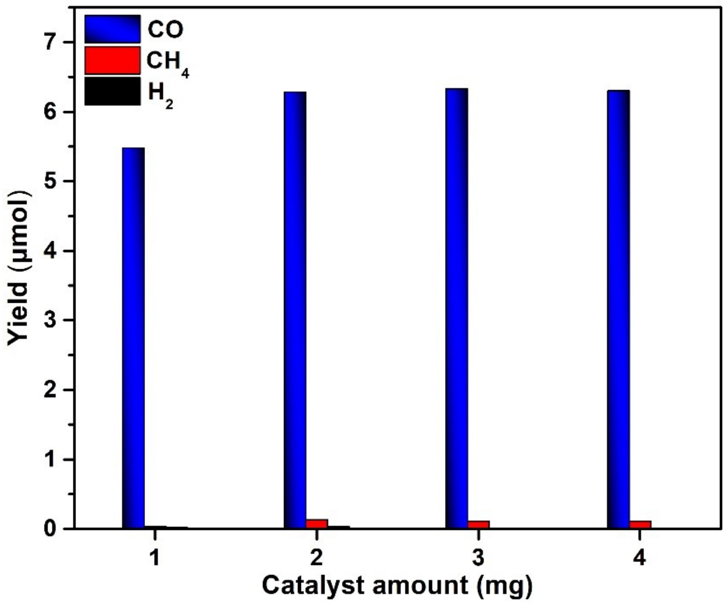

Figure S 9. Reaction conditions: 1Fe@f-gC_3N_4 (1-4 mg), TEA (0.2 mL), ACN (3.8 mL), Time = 18 h, light wavelength 427 nm, Reaction temp = 30˚C.

S2.6 Reusability of photocatalytic CO_2 reduction

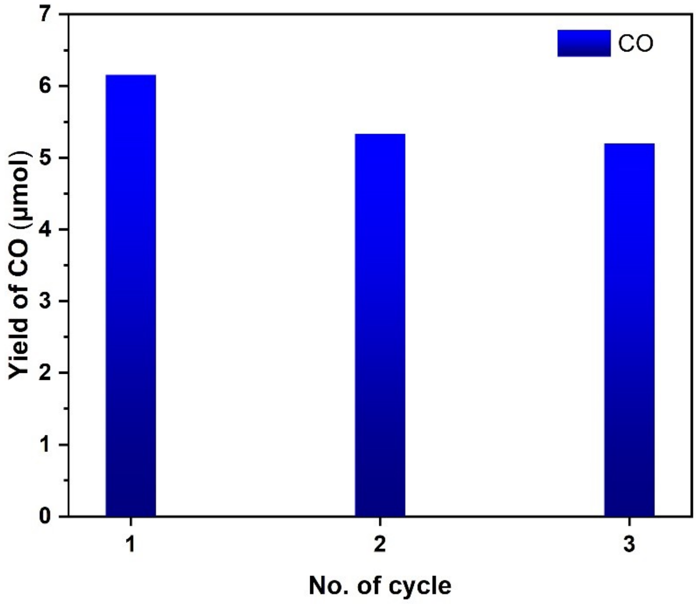

Figure S 10. Reaction conditions: 1Fe@f-gC_3N_4 (10 mg), TEA (0.2 mL), ACN (3.8 mL), Time = 18 h, light wavelength = 427 nm, Reaction temp = 30˚C.

S3. Catalyst Characterizations

S3.1 XRD measurements

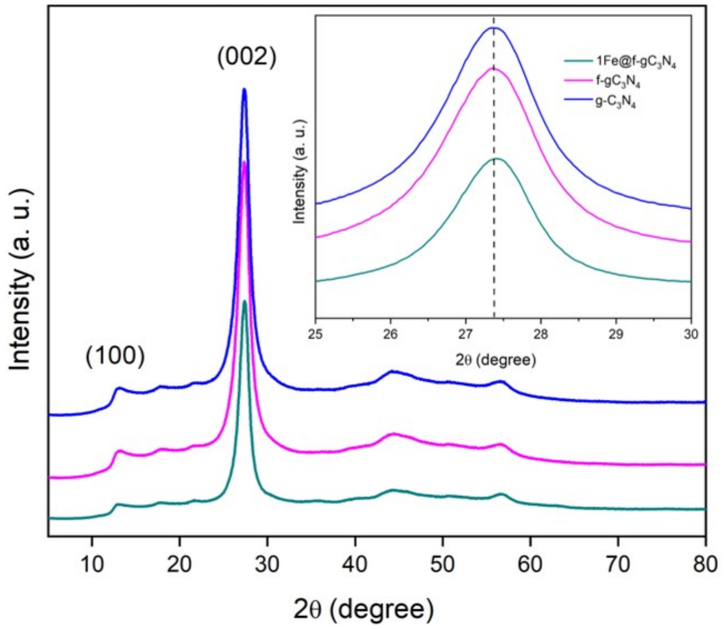

Figure S 11. XRD spectra of prepared gC_3N_4, f-gC_3N_4 and 1Fe@f-gC_3N_4.

S3.2 X-ray absorption spectroscopy

XAS spectra were recorded in transmission mode on the hard X-ray absorption beamline (CLAESS) of the ALBA CELLS synchrotron (Barcelona, Spain).[2] Synthesized 1Fe@f-gC_3N_4 and reference samples (FeOOH and Fe_2O_3) were mixed uniformly with cellulose and pressed in pellets to ensure the Fe *K*-edge X-ray absorption step close to 1 for references and ca. 0.2 for the sample. The samples were then loaded into a plastic sample holder, fixed with X-ray transparent Kapton tape, and mounted between the first and the second ionization chambers filled out with 48 % He and 52 % N_2, and with 12 % Kr and 88 % N_2, respectively. All spectra were acquired at room temperature by scanning a double-crystal Si (311) monochromator. The energy scale was calibrated with Fe foil. The absorption spectra were collected over an energy range of 6980−8270 eV in continuous mode. Five repeats were recorded for each sample to ensure reproducibility and then averaged to improve the signal-to-noise ratio.

The data was processed using the Athena program.[3] Scans were calibrated, aligned and normalized with background removal.

S3.3 Solid state NMR spectroscopy

Magic-angle-spinning (MAS) NMR experiments were performed at a magnetic field of 14.1 T (Larmor frequencies of 600.12, 150.92, 60.83, and 564.69 MHz for ^{1}H, ^{13}C, ^{15}N, and ^{19}F respectively) on a Bruker Avance-III spectrometer. The ^{1}H and ^{19}F MAS NMR spectra were acquired using a 1.3 mm probe head and a 60 kHz MAS rate. This acquisition involved a use of a rotor-synchronized, double-adiabatic spin-echo sequence with a 90° excitation pulse of 1.25 μs (2.30 μs for ^{19}F) followed by a pair of 50.0 μs tanh/tan short high-power adiabatic pulses (SHAPs) with 5 MHz frequency sweep.[4, 5] All pulses operated at the nutation frequency of 200 kHz (110 kHz for ^{19}F). 64 signal transients (10240 for ^{19}F) were acquired using a relaxation delay ranging from 0.1 to 5 s. Cross-polarization (CP) ^{1}H-^{13}C and ^{1}H-^{15}N CPMAS NMR spectra were recorded using a 7 mm probe head with a 7 kHz MAS rate, 65 kHz spinal

and 64 proton decoupling. For ^{1}H-^{13}C CPMAS acquisition, Hartmann-Hahn matched radiofrequency fields were applied for a contact interval of 1.5 ms and 2048 signal transients were collected using a relaxation delay of 5 s. The ^{1}H-^{15}N CPMAS acquisition involved contact interval of 5ms, and 16384 scans collected with relaxation time of 5 s. Chemical shifts are reported with respect to TMS (^{1}H, ^{13}C), nitromethane (^{15}N), and trichlorofluoromethane (^{19}F).

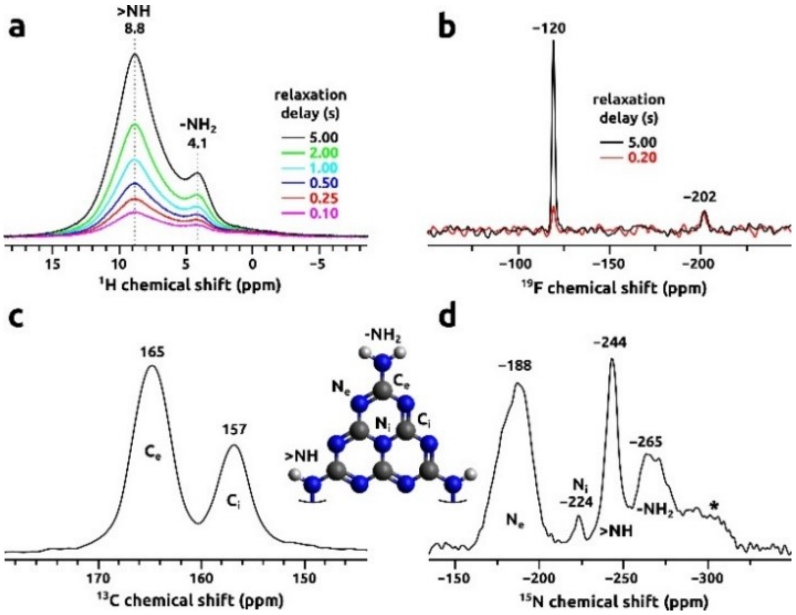

Figure S 12. ssNMR of solid-state ^{1}H MAS **(a)**, ^{19}F MAS **(b)**, ^{13}C CPMAS **(c)**, and ^{15}N CPMAS **(d)** spectra collected from the Fe@f-gC_3N_4 catalys'.

S3.4 XPS spectra of the fresh and reused samples

The surface composition was studied by X-ray photoelectron spectroscopy (XPS). The spectra were collected on a Prevac spectrometer equipped with a hemispherical VG SCIENTA R3000 analyzer (pass energy of 100 eV) and a monochromatized aluminum source AlKα (1486.6 eV). Binding energies were calibrated using the C 1s line at 284.8 eV. After a Shirley background subtraction, raw spectra were fitted using Gaussian-Lorentzian peak shapes in the Casa XPS software.

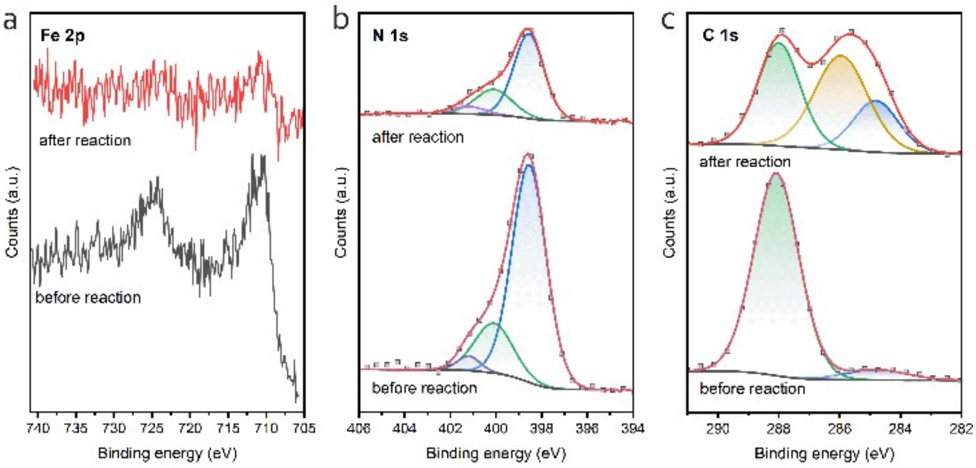

Figure S 13. XPS spectra recorded in the Fe 2p **(a)**, N 1s **(b)** and C 1s **(c)** regions for of 1Fe@f-gC_3N_4 and reused 1Fe@f-gC_3N_4.

S3.5 EPR spectroscopy

EPR measurements were conducted at RT using a Bruker EMX CW micro- X-band spectrometer (microwave frequency ≈ 9.87 GHz) equipped with an ER 4119HS-WI high-sensitivity optical resonator with a grid in the front side for irradiation the sample inside the EPR cavity. All the samples were irradiated by a Kessil lamp (λ = 427 nm) and measured by EPR before and after irradiation using a modulation frequency of 100 kHz.

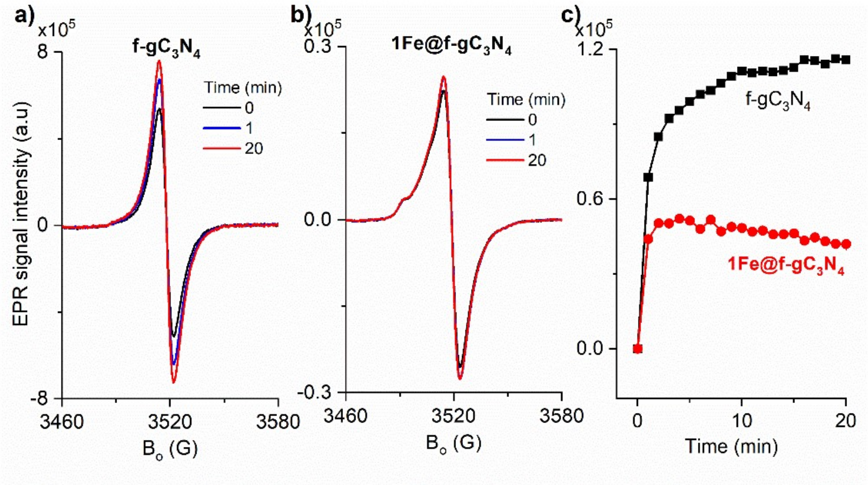

Figure S 14. EPR spectra of a) f-gC_3N_4; b) 1Fe@f-gC_3N_4 in the dark and during irradiation; c) the double integration of the EPR-active photoexcited electrons signal after subtraction of the corresponding spectrum in dark.

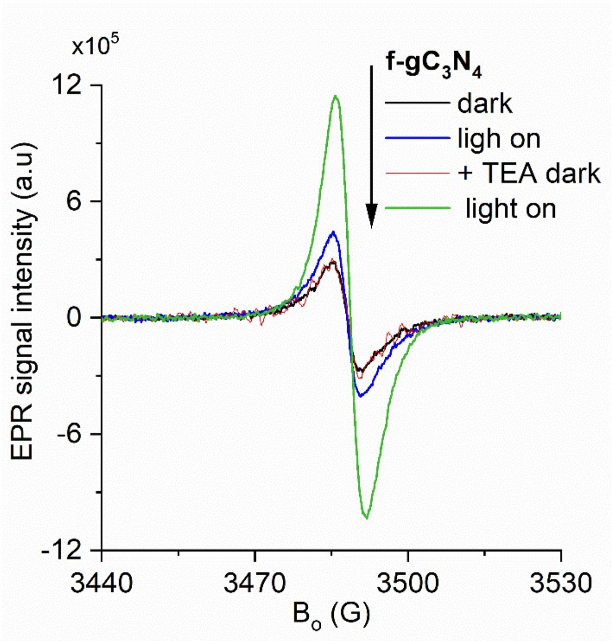

Figure S 15. EPR spectra of f-gC_3N_4 (15 mg) suspension in CH_3CN (0.5 ml) in the presence and absence of TEA (CH_3CN: TEA = 19:1) measured in the dark and during irradiation.

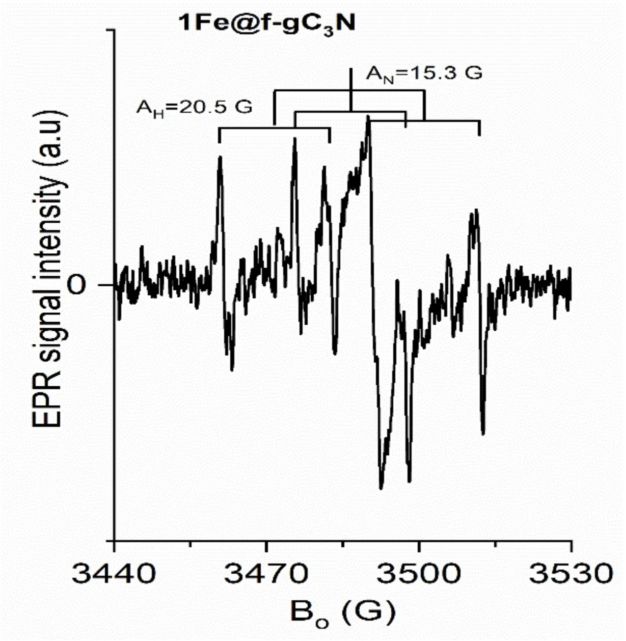

Figure S 16. EPR spectrum of DMPO-CO_2 spin adducts obtained over 1Fe@f-gC_3N_4 after substruction the EPR signal in the dark from that obtained during irradiation. Condition: 1mg1Fe@f-gC_3N_4 in 0.5 ml CH_3CN saturated with CO_2 for 5 min + 10µL DMPO.

S3.6 Photoluminescence Spectroscopy (PL)

Fluorescence spectroscopy

The emission spectra were recorded on an Edinburgh FLS980 under 365 nm excitation by a Xenon lamp.

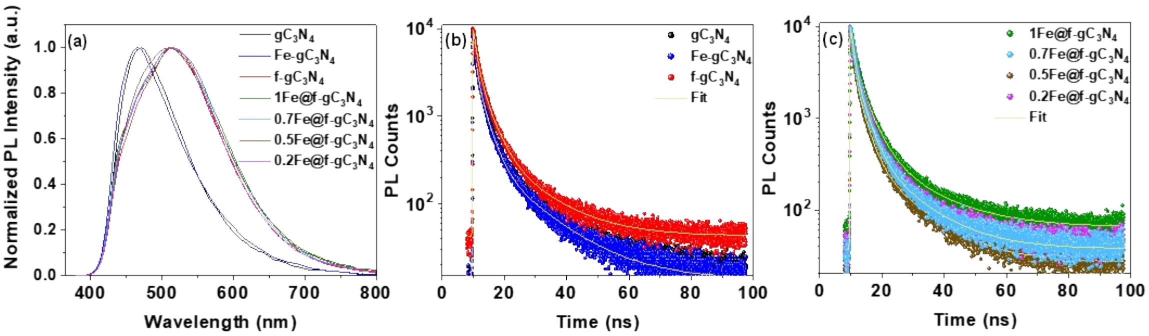

Figure S 17. Photoluminescence studies of the photocatalysts. **(a)** Emission spectra, and **(b,c)** lifetime profiles of gC_3N_4, Fe-gC_3N_4, and f-gC_3N_4 **(b)**, Fe-loaded f-gC_3N_4 photocatalysts **(c)** with their corresponding fit shown with a solid yellow line. The powdered samples for lifetime studies were excited at 360 nm with an excitation power intensity of 12 µW cm^{-2}.

The TCSPC is equipped with a pulsed laser, Nd: YAG (Quanta-Ray INDI-40, Spectra-Physics), and an optical parametric oscillator. The pulse duration is fixed at around 2 ps, and the time resolution of each experiment was about 100 ns. A beam splitter is used in the pathway to split the beam towards a photodiode to generate a start signal and excite the sample. The photons are collected, filtered, and focused on the entrance slit of a 30 cm focal length spectrograph (SpectroPro-300i Acton) and detected through a photomultiplier tube (Hamamatsu, R928). The transient electrical signal is then displayed by an oscilloscope

connected to the control computer. All the samples were excited at 360 nm with the excitation power intensity set at 12 μW cm^{-2} (0.2Fe@f-gC_3N_4 and f-gC_3N_4 were excited at the power of 4 μW cm^{-2} due to high photon counts). The decays were fitted using a tri-exponential fitting equation, and the average PL lifetimes (τ_{av}) were calculated using an intensity-weighted equation:

$$\tau_{avg} = \frac{\sum_{i}^{n} A_i \tau_i^2}{\sum_{i}^{n} A_i \tau_i}$$

Here, A_i is the amplitude fraction and τ_i is the fluorescence lifetime.

Table S 3 Fitting parameters for lifetime profiles of photocatalysts.

**Catalysts**	**τ_1 (ns)**	**τ_2 (ns)**	**τ_3 (ns)**	**A_1**	**A_2**	**A_3**	**τ_{av} (ns)**
gC_3N_4	0.7	3.0	14.2	0.64	0.32	0.03	5.2
Fe-gC_3N_4	0.7	2.9	13.8	0.65	0.32	0.03	5.0
f-gC_3N_4	1.0	3.5	13.5	0.61	0.35	0.04	5.2
1Fe@f-gC_3N_4	1.3	4.1	15.4	0.60	0.35	0.04	6.0
0.7Fe@f-gC_3N_4	1.0	3.2	13.0	0.59	0.36	0.04	5.0
0.5Fe@f-gC_3N_4	0.7	2.9	13.2	0.63	0.33	0.04	4.9
0.2Fe@f-gC_3N_4	1.0	3.4	13.2	0.59	0.37	0.04	5.2

S3.7 EIS Nyquist plot and photocurrent response curves.

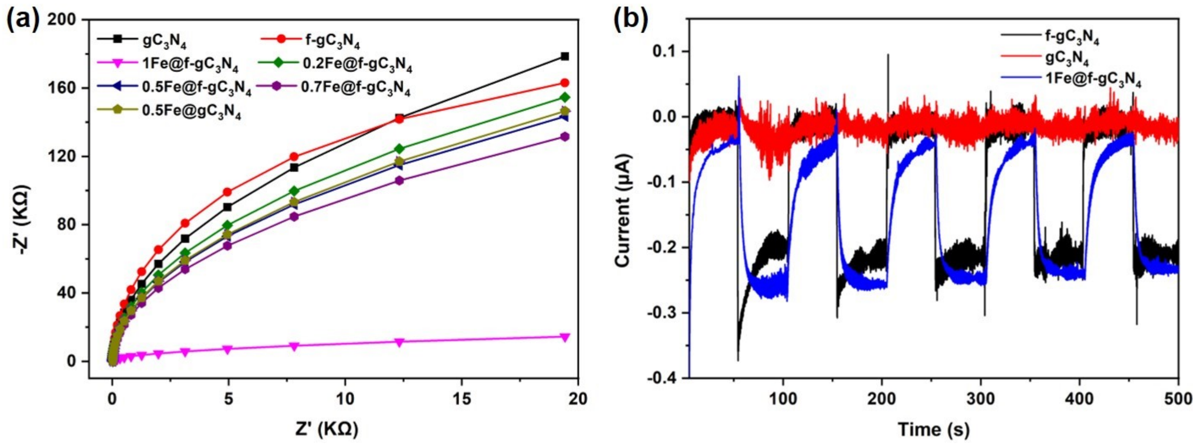

Figure S 18. (a) EIS Nyquist plot, (b) Photocurrent response curves of different catalysts.

Table S 4. Representative examples of reaction reported for photocatalytic CO_2 reduction.

Ent.	Catalyst	Solvent & Reductant	Light source (nm)	Prod. rate of CO ($\mu molg^{-1}h^{-1}$)	Selv. Of CO (%)	Ref.
1	WP-NC/g-C_3N_4 composite	CH_3CN (20 ml) + Distilled H_2O (0.5 ml)	300WXenon lamp	376	100	[6]
2	Co qpy@mpg-C_3N_4	BIH (0.05 M) +0.03M PhOH CH_3CN (3 ml)	400	7.98	98	[7]
3	Fe/mpg-C_3N_4	TEOA (0.8 ml) + CH_3CN (3.2 ml)	400W high pressure Hg lamp with $NaNO_2$ solution filter (≥ 400)	91.1	97	[8]
4	20%CPB-PCN composite	CH_3CN (30ml) + Deionized H_2O (0.1ml)	300WXe lamp (420)	149	100	[9]
5	g-C_3N_4/Au composite	CH_3CN: H_2O: TEOA (3 :1 :1)	Xenon lamp (320-780)	331.57	89.3	[10]
6	NH_2-MIL-101(Fe)/ g-C_3N_4-30 wt %	TEOA (2 mL)	300 W xenon arc lamp (400-780 nm filter)	22	100	[11]
7	g-C_3N_4, $CoCl_2$ (1 μmol), $Co(bpy)_3^{+2}$	CH_3CN (4 mL), TEOA (2 mL),	λ > 420nm.	37	86	[12]
8	In_2S_3-$CuInS_2$ (10 mg) +15 mg bipyridine (bpy) +4 μmol $CoCl_2$	CH_3CN: H_2O: TEOA (3:2:1)	300 W Xe lamp	19	100	[13]
9	BIF-20@g-C_3N_4	2 mL (CH_3CN: TEOA = 4:1)	300 W Xe lamp (400 nm UV-cut and 800 nm IR-cut filter)	53.869	77.6	[14]
10	UiO-66/CNNS	5 mL (TEOA:CH_3CN = 4:1)	300 W xenon arc lamp (400 nm < λ< 800 nm)	9.9	100	[15]
11	Feqpy-BA/g-C_3N_4	2 mL CH_3CN, 20 vol% TEA + 0.05M BIH	Blue led light (456 nm)	141.6	95.2	[16]
12	1Fe@f-g-C_3N_4 (1 mg)	4 mL (CH_3CN: TEA = 19:1)	427 nm kessil lamp	304	99	This work

S4. Theoretical calculation

Computational Details

To identify possible configurations of the Fe@f-gC_3N_4-CO_2 complex and reaction mechanisms leading to O_2 in acetonitrile (ACN), we used classical reactive molecular dynamics simulations (RMD)[17] based on a force field already tested [18] combined with DFT calculations. We used a polymeric carbon nitrides model consisting of fourteen melems with one f-substituent and three Fe ions (**Figure S19**). We surrounded the model with 886 ACN and $^{16}CO_2$ molecules and placed some CO_2 close to the metal centers to favor adsorption. The simulation box (33x35x12 Å^3) was replicated in all directions (**Figure S20**). RMD equilibration simulations were carried out in the NVT/NPT ensembles at ambient temperature and pressure (298 K and 1 atm). Production trajectories were performed in the NVT ensemble, and the system configurations were sampled every 0.01 ps. The ReaxFF version, implemented in the Amsterdam Density Functional (ADF)/ReaxFF 2023.1 package, was used for all the simulations. The time step was 0.2 fs, and the temperature was controlled through the Hoover-Nosé thermostat with a relaxation constant of 0.1 ps. No constraints were imposed on the system.

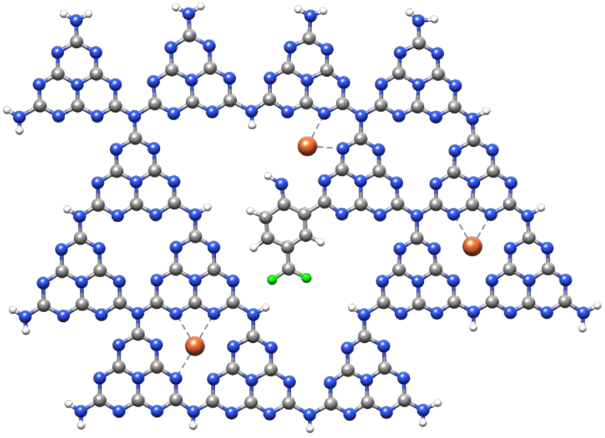

Figure S 19. Initial catalyst structure used for the ReaxFF MD simulations. Ball and stick model Color code: C gray, N blue, F green, Fe orange, H white. The Fe ions maintained their chelated arrangements between the nitrogens of adjacent triazine rings.

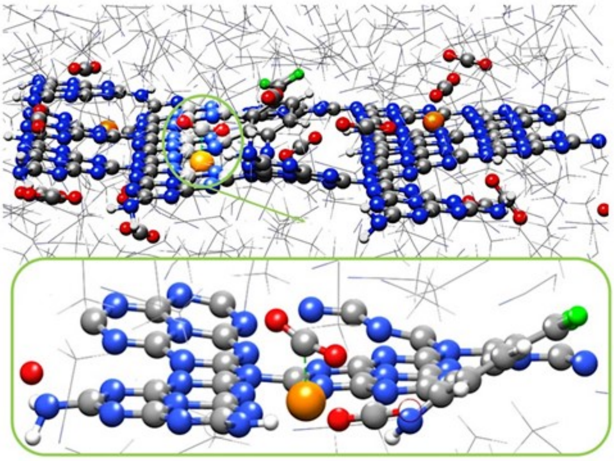

Figure S 20. Top. Perturbed RMD: catall structure surrounded by ACN (grey lines) and CO_2 (ball and stick model) molecules. Color code: C gray, N blue, F green, Fe orange, O red, H

white. The Fe ions maintained their chelated arrangements between the nitrogens of adjacent triazine rings. **Bottom**. Magnified view of the site where one CO_2 molecule was chemisorbed.

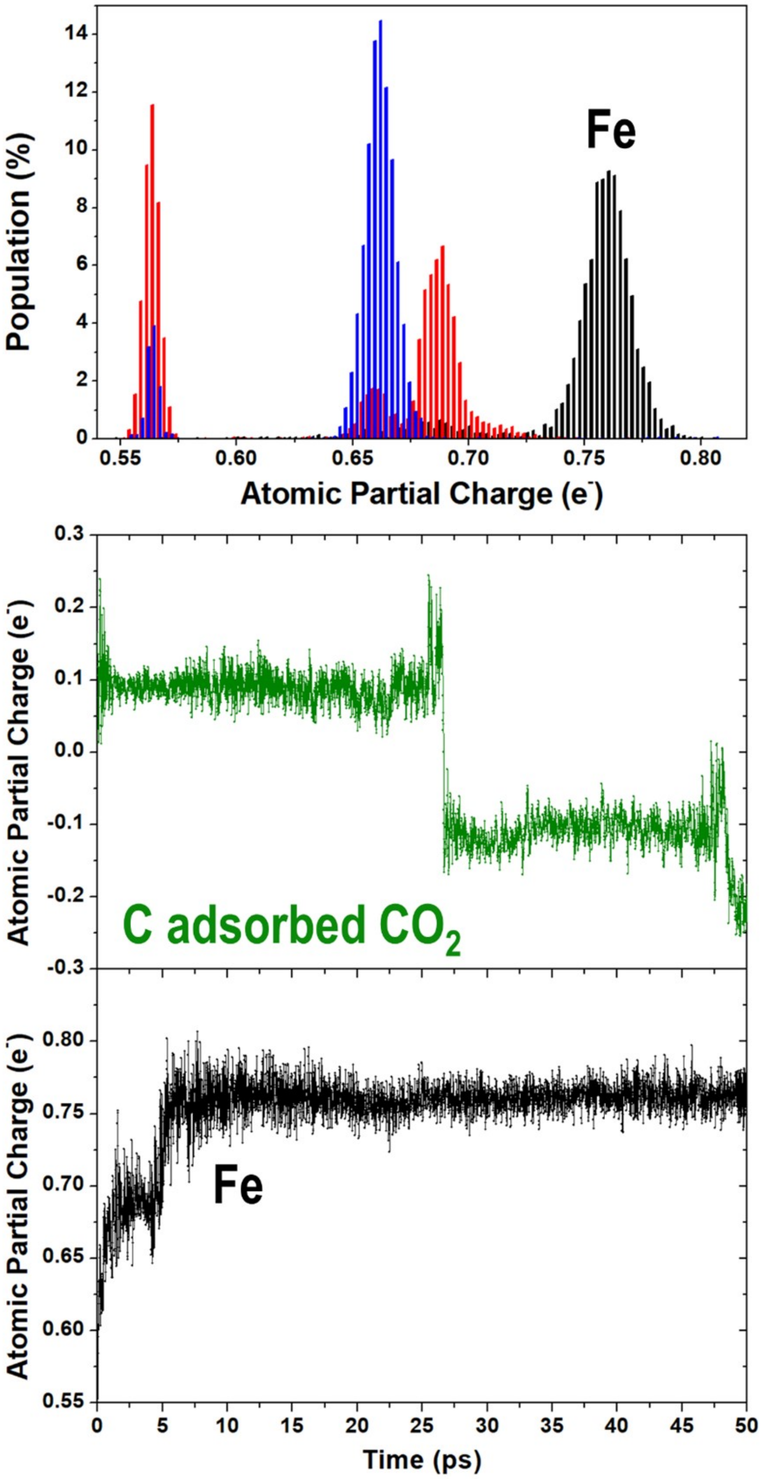

Figure S 21. Top. Distributions of the atomic partial charges of all the Fe atoms during the perturbed RMD simulations. The black plot corresponds to the Fe atom with a CO_2 molecule coordinated through its carbon (**Figure S20**). The evolution of the charge is shown in the bottom plot. **Bottom**. Evolution of the partial charges of the carbon atom of the adsorbed CO_2 (green line) and the connected Fe (black line).

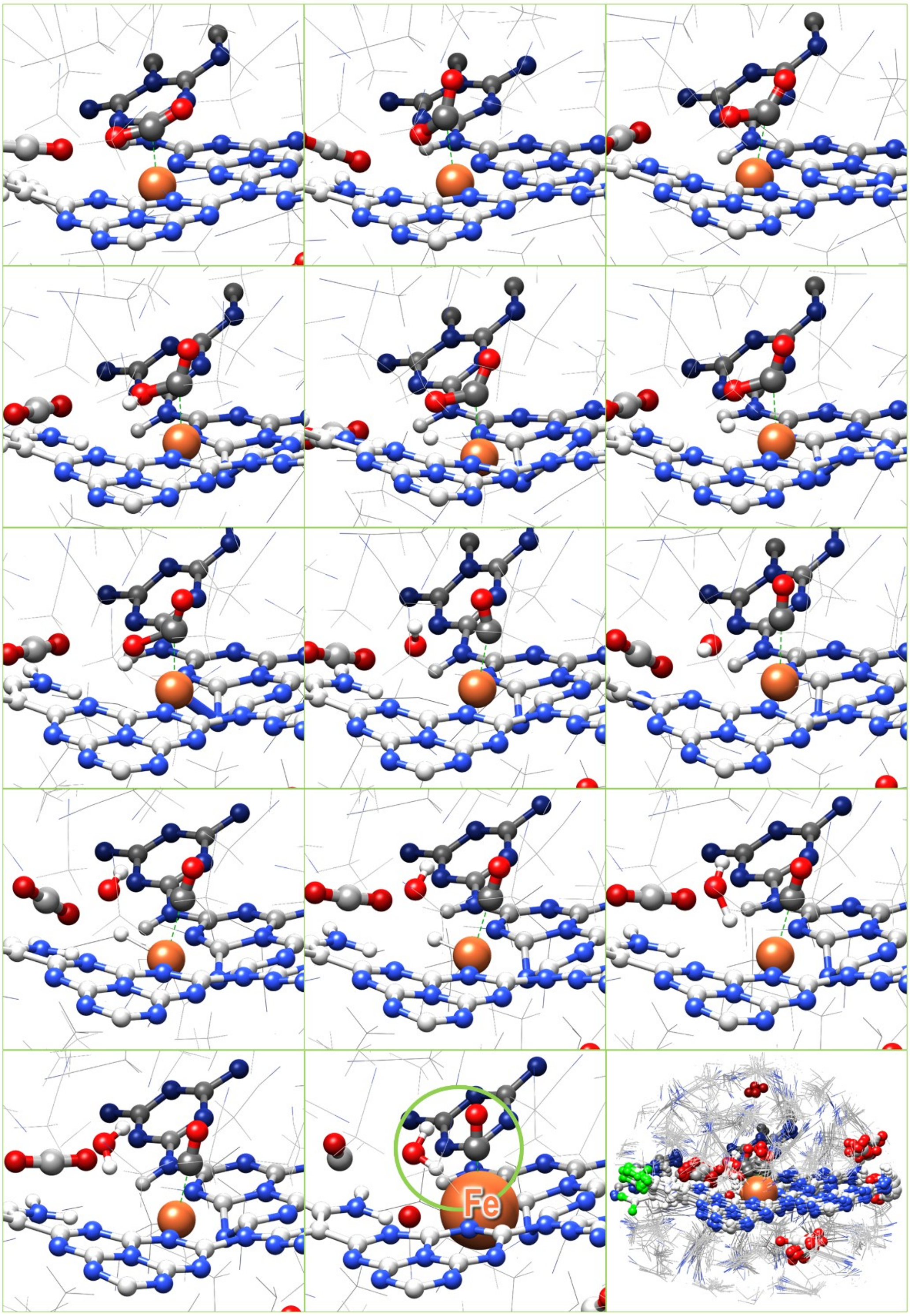

Figure S 22. Representative Snapshots of the CO_2 reduction mechanism disclosed by the perturbed RMD simulations. ACN (grey lines) and melem/CO_2 molecules (ball and stick model). Color code: C gray, N blue, F green, Fe orange, O red, H white.

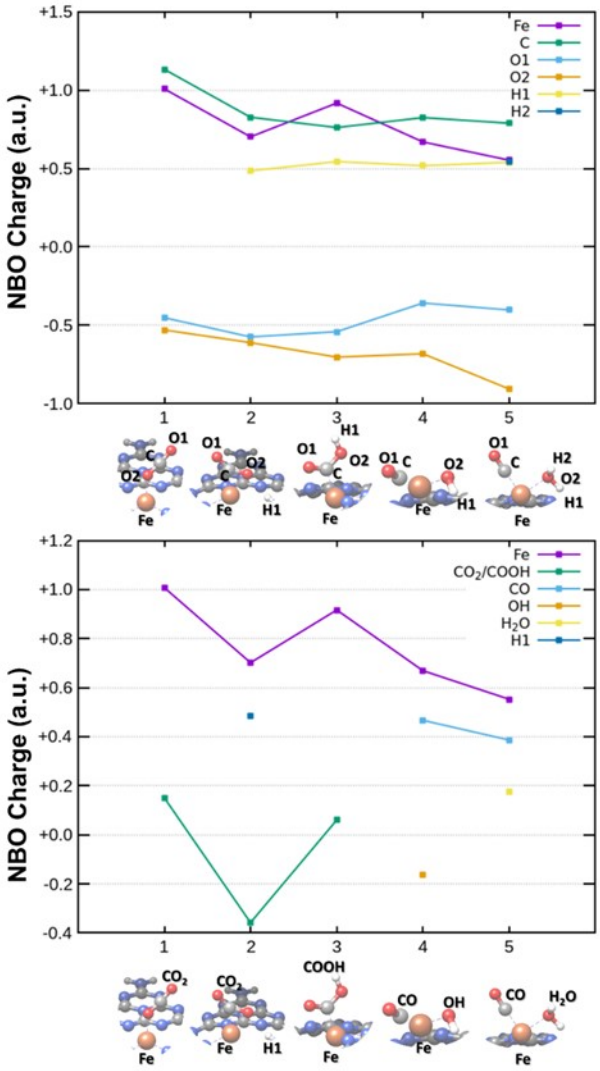

Figure S 23. NBO charge analysis of states 1-5 is shown in **Fig. 5** according to a partition in single atoms (a) or aggregated form (b).

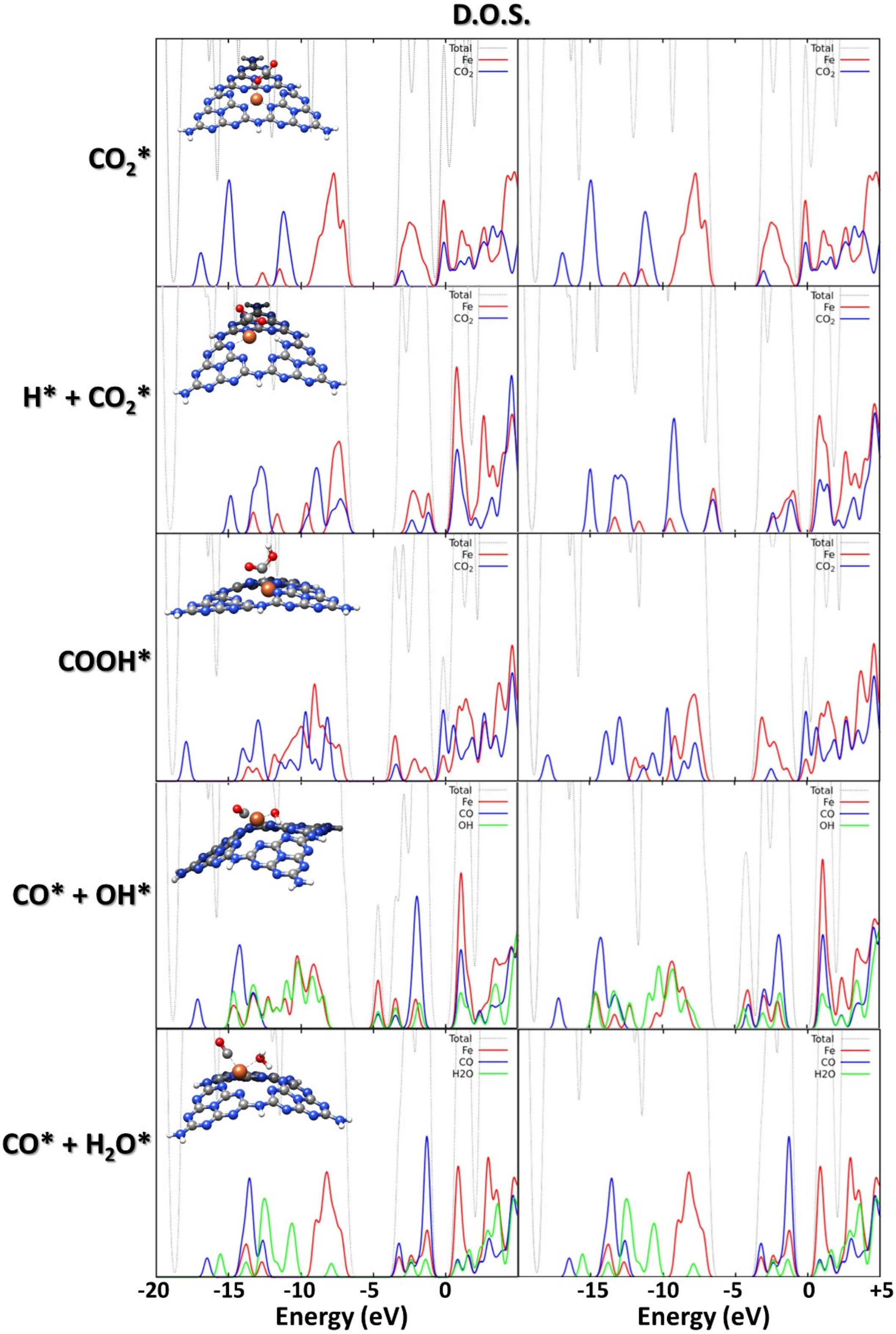

Figure S 24 Total and Projected Density of States (DOS) corresponding **to Spin Up (left column) and Spin Down (right column) of** states 1-5 of the high-coordination site shown in **Fig. 5** of the main article (structures are also reported as in-set pictures).

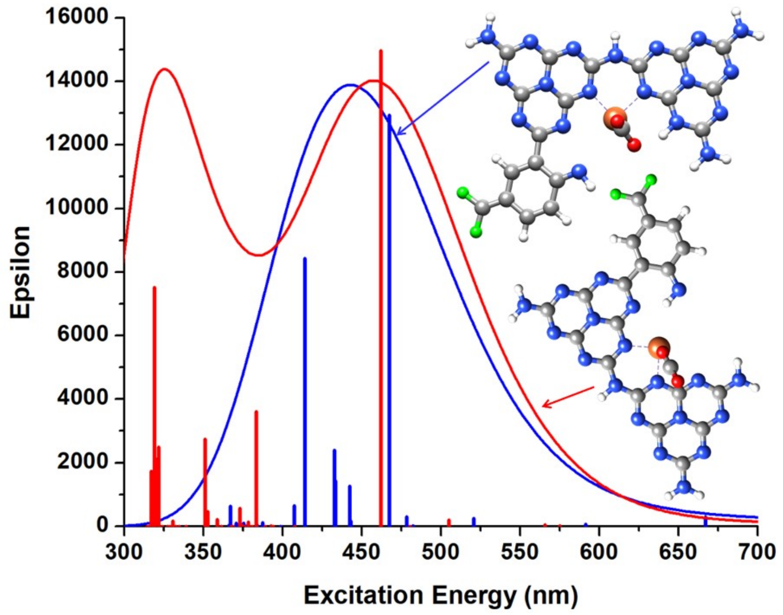

Figure S 25. Simulated UV-vis spectra of the complex minimum energy structures. Color code: C gray, N blue, F green, Fe orange, O red, H white.

References

1. T. Zhang, W. Schilling, S. U. Khan, H. Y. V. Ching, C. Lu, J. Chen, A. Jaworski, G. Barcaro, S. Monti, K. De Wael, A. Slabon and S. Das, Atomic-Level Understanding for the Enhanced Generation of Hydrogen Peroxide by the Introduction of an Aryl Amino Group in Polymeric Carbon Nitrides, *ACS Catal.*, 2021, **11**, 14087-14101.
2. L. Simonelli, C. Marini, W. Olszewski, M. Ávila Pérez, N. Ramanan, G. Guilera, V. Cuartero, K. Klementiev and N. L. Saini, CLAESS: The hard X-ray absorption beamline of the ALBA CELLS synchrotron, *Cogent Phys.*, 2016, **3**.
3. B. Ravel and M. Newville, ATHENA, ARTEMIS, HEPHAESTUS: data analysis for X-ray absorption spectroscopy using IFEFFIT, *J. Synchrotron. Radiat.*, 2005, **12**, 537-541.
4. G. Kervern, G. Pintacuda and L. Emsley, Fast adiabatic pulses for solid-state NMR of paramagnetic systems, *Chem. Phys. Lett.*, 2007, **435**, 157-162.
5. T. L. Hwang, P. C. van Zijl and M. Garwood, Fast broadband inversion by adiabatic pulses, *J. Magn. Reson.*, 1998, **133**, 200-203.
6. X. Zhang, J. Yan, F. Zheng, J. Zhao and L. Y. S. Lee, Designing charge transfer route at the interface between WP nanoparticle and g-C_3N_4 for highly enhanced photocatalytic CO_2 reduction reaction, *Appl. Catal. B: Environ.*, 2021, **286**, 119879.
7. B. Ma, G. Chen, C. Fave, L. Chen, R. Kuriki, K. Maeda, O. Ishitani, T. C. Lau, J. Bonin and M. Robert, Efficient Visible-Light-Driven CO_2 Reduction by a Cobalt Molecular Catalyst Covalently Linked to Mesoporous Carbon Nitride, *J. Am. Chem. Soc.*, 2020, **142**, 6188-6195.
8. C. Cometto, R. Kuriki, L. Chen, K. Maeda, T. C. Lau, O. Ishitani and M. Robert, A Carbon Nitride/Fe Quaterpyridine Catalytic System for Photostimulated CO_2-to-CO Conversion with Visible Light, *J. Am. Chem. Soc.*, 2018, **140**, 7437-7440.
9. M. Ou, W. Tu, S. Yin, W. Xing, S. Wu, H. Wang, S. Wan, Q. Zhong and R. Xu, Amino-Assisted Anchoring of $CsPbBr_3$ Perovskite Quantum Dots on Porous g-C_3N_4 for Enhanced Photocatalytic CO_2 Reduction, *Angew. Chem. Int. Ed. Engl.*, 2018, **57**, 13570-13574.
10. Q. Wang, Z. Fang, X. Zhao, C. Dong, Y. Li, C. Guo, Q. Liu, F. Song and W. Zhang, Biotemplated g-C_3N_4/Au Periodic Hierarchical Structures for the Enhancement of Photocatalytic CO_2 Reduction with Localized Surface Plasmon Resonance, *ACS Appl. Mater. Interfaces*, 2021, **13**, 59855-59866.
11. X.-Y. Dao, X.-F. Xie, J.-H. Guo, X.-Y. Zhang, Y.-S. Kang and W.-Y. Sun, Boosting Photocatalytic CO_2 Reduction Efficiency by Heterostructures of NH2-MIL-101(Fe)/g-C_3N_4, *ACS Appl. Energy Mater.*, 2020, **3**, 3946-3954.
12. J. Lin, Z. Pan and X. Wang, Photochemical Reduction of CO_2 by Graphitic Carbon Nitride Polymers, *ACS Sustain. Chem. Eng.*, 2014, **2**, 353-358.
13. J. Yang, X. Zhu, Z. Mo, J. Yi, J. Yan, J. Deng, Y. Xu, Y. She, J. Qian, H. Xu and H. Li, A multidimensional In_2S_3–$CuInS_2$ heterostructure for photocatalytic carbon dioxide reduction, *Inorg. Chem. Front.*, 2018, **5**, 3163-3169.
14. G. Xu, H. Zhang, J. Wei, H. X. Zhang, X. Wu, Y. Li, C. Li, J. Zhang and J. Ye, Integrating the g-C_3N_4 Nanosheet with B-H Bonding Decorated Metal-Organic Framework for CO_2 Activation and Photoreduction, *ACS Nano*, 2018, **12**, 5333-5340.
15. L. Shi, T. Wang, H. Zhang, K. Chang and J. Ye, Electrostatic Self-Assembly of Nanosized Carbon Nitride Nanosheet onto a Zirconium Metal–Organic Framework for Enhanced Photocatalytic CO_2 Reduction, *Adv. Funct. Mater.*, 2015, **25**, 5360-5367.
16. Y. Wei, L. Chen, H. Chen, L. Cai, G. Tan, Y. Qiu, Q. Xiang, G. Chen, T. C. Lau and M. Robert, Highly Efficient Photocatalytic Reduction of CO_2 to CO by In Situ Formation of a Hybrid Catalytic System Based on Molecular Iron Quaterpyridine Covalently Linked to Carbon Nitride, *Angew. Chem. Int. Ed. Engl.*, 2022, **61**, e202116832.
17. ReaxFF 2023.1, SCM, Theoretical Chemistry, Vrije Univer-siteit, Amsterdam, The Netherlands, http://www.scm.com, ReaxFF 2023.2021, SCM, Theoretical Chemistry, Vrije Univer-siteit, Amsterdam, The Netherlands, http://www.scm.com.
18. P. Ren, T. Zhang, N. Jain, H. Y. V. Ching, A. Jaworski, G. Barcaro, S. Monti, J. Silvestre-Albero, V. Celorrio, L. Chouhan, A. Rokicinska, E. Debroye, P. Kustrowski, S. Van Doorslaer, S. Van Aert, S. Bals and S. Das, An Atomically Dispersed Mn-Photocatalyst for Generating Hydrogen Peroxide from Seawater via the Water Oxidation Reaction (WOR), *J. Am. Chem. Soc.*, 2023, **145**, 16584-16596.
